# Performance of epistasis detection methods in semi-simulated GWAS

**DOI:** 10.1186/s12859-018-2229-8

**Published:** 2018-06-18

**Authors:** Clément Chatelain, Guillermo Durand, Vincent Thuillier, Franck Augé

**Affiliations:** 1 SANOFI R&D, Translational Sciences, Chilly Mazarin, 91385 France; 2 Laboratoire de Probabilités et Modèles Aléatoires, Université Pierre et Marie Curie, 4, place Jussieu, Paris Cedex 05, 75252 France; 3 SANOFI R&D, Biostatistics & Programming, Chilly Mazarin, 91385 France

**Keywords:** Genome-wide association studies, Epistasis, Simulation

## Abstract

**Background:**

Part of the missing heritability in Genome Wide Association Studies (GWAS) is expected to be explained by interactions between genetic variants, also called epistasis. Various statistical methods have been developed to detect epistasis in case-control GWAS. These methods face major statistical challenges due to the number of tests required, the complexity of the Linkage Disequilibrium (LD) structure, and the lack of consensus regarding the definition of epistasis. Their limited impact in terms of uncovering new biological knowledge might be explained in part by the limited amount of experimental data available to validate their statistical performances in a realistic GWAS context. In this paper, we introduce a simulation pipeline for generating real scale GWAS data, including epistasis and realistic LD structure. We evaluate five exhaustive bivariate interaction methods, fastepi, GBOOST, SHEsisEpi, DSS, and IndOR. Two hundred thirty four different disease scenarios are considered in extensive simulations. We report the performances of each method in terms of false positive rate control, power, area under the ROC curve (AUC), and computation time using a GPU. Finally we compare the result of each methods on a real GWAS of type 2 diabetes from the Welcome Trust Case Control Consortium.

**Results:**

GBOOST, SHEsisEpi and DSS allow a satisfactory control of the false positive rate. fastepi and IndOR present an increase in false positive rate in presence of LD between causal SNPs, with our definition of epistasis. DSS performs best in terms of power and AUC in most scenarios with no or weak LD between causal SNPs. All methods can exhaustively analyze a GWAS with 6.10^5^ SNPs and 15,000 samples in a couple of hours using a GPU.

**Conclusion:**

This study confirms that computation time is no longer a limiting factor for performing an exhaustive search of epistasis in large GWAS. For this task, using DSS on SNP pairs with limited LD seems to be a good strategy to achieve the best statistical performance. A combination approach using both DSS and GBOOST is supported by the simulation results and the analysis of the WTCCC dataset demonstrated that this approach can detect distinct genes in epistasis. Finally, weak epistasis between common variants will be detectable with existing methods when GWAS of a few tens of thousands cases and controls are available.

**Electronic supplementary material:**

The online version of this article (10.1186/s12859-018-2229-8) contains supplementary material, which is available to authorized users.

## Backgrounds

During the past decade, Genome-Wide Association Studies (GWAS) have focused on individual Single Nucleotide Polymorphisms (SNPs), looking at variants exhibiting independent, additive and cumulative effects on phenotypes. To date thousands of SNPs have been associated with diseases and other complex traits [1], but in most cases those variants independently explain only a small fraction of the estimated heritability [2, 3]. For example, in Crohn’s Disease, cumulative additive effects explain 10.6% of the variability while the estimated heritability is 53% in Type-2 diabetes, 4.7% for an estimated heritability of 26% and in Lupus, 6.6% for an estimated heritability of 44% [4]. The correct estimation of this missing heritability is still a subject of research interest [5] which may be due to a limitation of this additive model, confirming geneticist’s hypothesis that most phenotypes are not only driven by genetic variants acting independently, but that other phenomenons have to be taken into account including, but not limited to: epigenetics, environment interactions, and genetic interactions between loci (epistasis) [6]. Zuk et al. [7] evaluated that up to 80% of the missing heritability could be due to epistasis in some diseases. Genome Wide scan for epistasis are therefore seen as potentially a very fruitful approach to better understand the genetics of these diseases and to identify new therapeutic strategies.

A vast number of methods for detecting epistasis have been developed in recent years, ranging from exhaustive bivariate tests to machine learning methods (for recent reviews see [8, 9]). Exhaustive bivariate methods (referred to as bivariate methods hereafter) test all pairs of SNPs in a case control GWAS for epistasis that we define as departure from additivity on a logit scale [10]. These methods are adapted to a full analysis of GWAS data, because require less computation than other methods such as random forests and they might be less impacted by high dimensionality. Moreover bivariate methods do not introduce prior biological knowledge, such as network methods, which may limit the discovery of new biology. Finally, we note that epistasis definitions are not always equivalent to other types of approaches, such as Random Forest. The present paper focuses therefore on a selection of bivariate methods. The performance of other types of methods will be evaluated in future work. Bivariate methods face major technical challenges due to the vast number of combinations to be tested even for pairwise analysis (billions to trillions for a typical GWAS): controlling the number of false positive while maintaining sufficient power and ensuring computing time remains acceptable.

The direct evaluation of the statistical performance of these methods on real GWAS is not possible, as true epistatic interactions underlying complex diseases are still largely unknown. In contrast the power and false positive rate of these methods can be easily evaluated in simulated GWAS where the phenotype-genotype relationship is predefined. Evaluations using different types of simulations have been performed. Wang et al. [11] and Frost el al. [12] compared their methods on simulated datasets with independant SNPs. Emily [13], Goudey et al. [14], and Yu et al. [15] compared their methods to PLINK, *χ*^2^, and BOOST on simulated contingency tables with two SNPs including linkage disequilibrium (LD). Wan et al. [16] compared BOOST and PLINK under *H*_0_ using evolutionary simulations [17] to generate datasets of 38836 SNPs with simulated LD. Other studies also evaluated the performance of Gene-based bivariate methods [18, 19] or interaction tests for quantitative traits [20] in simulated GWAS. Some of these endeavours have in particular highlighted the influence of LD on the control of the type 1 error rates (see [20] for instance). However, the previous simulations present generally two important simplifications compared to real GWAS: (i) the absence of realistic, population specific LD pattern between simulated SNPs and (ii) the limited number of SNPs compared to a real GWAS. LD presents a complex structure at the genome-wide scale and accurate evaluation of the false positive rate of exhaustive bivariate methods would therefore require simulation of such realistic data. Moreover, most comparisons includes few scenarios, less than three methods and are performed in the context of papers introducing a new method to highlight its performances. There is therefore a lack of independent and exhaustive comparisons of these methods.

Several approaches have been implemented to simulate case control GWAS on a genome wide scale with realistic LD using a reference panel: HAPGEN2 [21], GenomeSIMLA [17], GWASIMULATOR [22], and waffect [23]. For instance Spencer et al. [24] evaluated the power of the univariate *χ*^2^ test with various SNP chips on genome wide HAPGEN simulations. Perduca et al. [23] evaluated the power of univariate tests in PLINK on waffect simulations including more than 10^5^ SNPs. However, no study evaluating the performance of epistatic detection softwares with these approaches has been reported, even if all three approaches have the capacity to simulate epistatic interactions. Because GWASIMULATOR is not able to simulate epistasic interactions between SNPs on a same chromosome, and therefore in LD, we introduce a new simulation pipeline combining the approaches of GWASIMULATOR [22] and waffect [23].

The objective of the present work is to provide an evaluation of the performance of a set of representative epistasis detection methods in simulated GWAS with features close to a real GWAS, both in terms of size and LD pattern. Five methods are selected due to their ability to scale for exhaustive genome wide bivariate analysis with a GPu implementation, their performances in previous studies, their popularity, and their representation of different approaches (LD, regression, Haplotype, ROC curve). Fastepi [25] is implemented in the software PLINK (option –fast-epistasis) and used as a fast method to test for interactions. It is based on a 2×2 contingency table of allele counts and tests a SNP pair for epistasis by comparing their LD in cases and controls. SHEsisEpi [26] is another LD based method based on a 3×3 contingency table. Both methods use a *χ*^2^ statistics with one degree of freedom and may therefore be more powerful than other approaches. IndOR [13] is also based on the correlation between two SNPs in cases and controls, inspired by a biological definition of epistasis, the effect of one gene masking the effect of another. GBOOST [27] compares two regression models with or without an interaction term using a likelihood ratio test to detect epistasis. GBOOST corresponds to Ficher’s definition of epistasis and has been used as benchmark method in several studies as discussed above. Finally DSS [14] is a model free approach based on the ROC curve to test the improvement of the discriminative power when using both SNPs together or independently. For each method the following performances are evaluated: power, false positive rate control, and computational performance. This article presents firstly a simulation pipeline for generating semi-simulated GWAS with realistic LD and epistasis, and then the performance comparison of the five methods. Finally the results of each method applied to a GWAS of Type 2 Diabetes (T2D) from the Welcome Trust Case Control Consortium [28] are presented.

## Methods

### Three-step GWAS simulation

We consider a set of template genotypes $\boldsymbol {X}_{i}\in \mathbb {R}^{p}$, *i*∈{1,..,*n*}, with $X_{ij_{c}}\in \{0,1,2\}$ representing for sample *i* the number of minor alleles at locus *j*_*c*_ of chromosome *c*∈{1,..,*C*}, with *p*_*c*_ the number of loci on chromosome *c* and $p={\sum \nolimits }_{c=1}^{C} p_{c}$ the total number of loci.

For step one a population of *m* individuals (*m*≫*n*) with genotype reproducing the LD structure of the template genotypes is simulated following the method of Li et al. [22]: for each simulated genotype *k*∈{1,..,*m*} and for each chromosome *c*, (i) a start locus *d*_*c*_ is selected uniformly at random, (ii) a (2*l*+1)-SNP haplotype [*d*_*c*_−*l*,*d*_*c*_+*l*]∈{0,1,2}^2*l*+1^ is sampled uniformly from the template genotypes, (iii) the right part of the chromosome is generated by choosing the allele at locus *d*_*c*_+*i* randomly among template genotypes corresponding to the simulated haplotype at loci [*d*_*c*_−2*l*+*i*,*d*_*c*_−1+*i*] for *l*<*i*≤*p*_*c*_−*d*_*c*_, (iv) similarly the left part of the chromosome is generated by choosing the alleles at loci *d*_*c*_−*i* given the simulated haplotype at [*d*_*c*_+1−*i*,*d*_*c*_+2*l*−*i*] for *l*<*i*<*d*_*c*_. In the following we use the European (EUR) samples from the 1000 Genomes Project phase 3 [29] as template genotypes and 129,238 SNP markers selected from the Affymetrix 500K chip to generate a population of 100,000 female samples. Markers with low MAF (< 0.05) or not in Equilibrium of Hardy Weinberg (*p*<10^−3^) in the template genotypes were excluded. This step allows the accurate replication of the LD structure of the EUR group for an accurate evaluation of the false positive rate in the present benchmark.

For step two we consider a set *I* of SNPs causal for a disease *D*, with a disease probability *p*_*k*_=*P*(*D*|*G*_*k*_) given a genotype *G*_*k*_ described with a logit model:

1 $$  \log\left(\frac{p_{k}}{1-p_{k}}\right) = \alpha + \underbrace{\sum\limits_{x\in I} \boldsymbol{\beta}_{x} \boldsymbol{1}_{x_{k}}}_{\textrm{main effect}} + \underbrace{\sum\limits_{(x,y)\in I^{2}} \boldsymbol{1}_{x_{k}}^{\mathsf{T}} \boldsymbol{\beta}_{x,y} \boldsymbol{1}_{y_{k}}}_{\textrm{\(2^{nd}\) order epistasis}}  $$

with *α* the intercept, $\boldsymbol {\beta }_{x}\in \mathbb {R}^{2}$ the main effect parameter vector for SNP *x*, $\boldsymbol {\beta }_{x,y} \in \mathbb {R}^{2 \times 2}$ the matrix of interaction coefficients between SNPs *x* and *y*, and $\boldsymbol {1}_{x_{k}} \in \{0,1\}^{2}$ the indicator vector for the value of SNP *x* in sample *k*. In the present work we restrict SNP interactions to the second order and to disease models with two causal SNPs (*a*,*b*). However the simulation procedure can be easily generalized to higher order interactions by adding the corresponding terms in Eq. 1. Each disease scenario is defined by the following parameters: (i) prevalence *K*=0.15, (ii) MAF *f*_*a*_ and *f*_*b*_ of each causal SNP, (iii) LD *r*^2^ between SNP *a* and *b*, (iv) marginal risk ratios ***R***_*a*_=(*r*_*a*_,*r*_*a*_)^T^ and ***R***_*b*_=(*r*_*b*_,*r*_*b*_)^T^ (only dominant models were considered), and (v) epistasis model matrix ***ρ*** parametrized as departure from product relative risk, with a risk ratio given by $\boldsymbol {R}_{a}^{\mathsf {T}} \boldsymbol {\rho } \boldsymbol {R}_{b}$. The disease prevalence *K*=0.15 represents a high estimate for a complex disease such as diabetes (estimated *K*=0.12 according to the American Diabetes Association) or NAFLD (estimated *K*=0.13 [30]). The disease model is constructed as follows: (i) the SNP pair best satisfying the MAF and LD constraints is selected through an exhaustive genome-wide search, (ii) the disease model (Eq. 1) is solved numerically to satisfy the prevalence and risk ratio constraints. The 234 scenarios considered are summarized in Table 1.

**Table 1:** Two hundred thirty four disease scenarios considered in the simulations

Epistasis model ***ρ***	MAF (*f*_*a*_,*f*_*b*_)	LD *r*^2^	Main effect (*r*_*a*_,*r*_*b*_)	Interaction *ρ*	# scenarios
$M_{0} = \left (\begin {array}{lll} 1 & 1 & 1 \\ 1 & 1 & 1 \\ 1 & 1 & 1\\ \end {array}\right)$	(0.15,0.15) (0.3,0.3)	0,0.2,0.5	(1,1) (1.5,1.5) (1,1.5)	1	18
$M_{1} = \left (\begin {array}{lll} 1 & 1 & 1 \\ 1 & \rho & \rho \\ 1 & \rho & \rho \end {array}\right)$	(0.15,0.15) (0.3,0.3)	0,0.2,0.5	(1,1) (1.5,1.5) (1,1.5)	2, 3, 5	54
$M_{2} = \left (\begin {array}{lll} 1 & 1 & 1 \\ 1 & 1 & 1 \\ 1 & 1 & \rho \\ \end {array}\right)$	(0.15,0.15) (0.3,0.3)	0,0.2,0.5	(1,1) (1.5,1.5) (1,1.5)	2, 5, 10	54
$M_{3} = \left (\begin {array}{lll} 1 & 1 & 1 \\ 1 & \rho & \rho ^{2} \\ 1 & \rho ^{2} & \rho ^{4} \end {array}\right)$	(0.15,0.15) (0.3,0.3)	0,0.2,0.5	(1,1) (1.5,1.5) (1,1.5)	2, 3, 5	54
$M_{4} = \left (\begin {array}{lll} 1 & 1 & 1 \\ 1 & 1 & \rho \\ \rho & \rho & \rho \\ \end {array}\right)$	(0.15,0.15) (0.3,0.3)	0,0.2,0.5	(1,1) (1.5,1.5) (1,1.5)	2, 3, 5	54
					234

Each scenario includes two causal SNPs *a* and *b* with MAF *f*_*a*_ and *f*_*b*_ respectively, and with a LD *r*^2^. The relative risk of genotype (*a*,*b*)=(*i*,*j*) vs genotype (0,0) is given by *R*_*i*,*j*_=*r*_*a*,*i*_*r*_*b*,*j*_*ρ*_*i*,*j*_. The matrix *ρ*_*i*,*j*_ is given by the epistasis model (null, dominant-dominant, recessive-recessive, multiplicative or alternative from top to bottom) and the scalar interaction parameter *ρ*. The main effect for SNP *a* is *r*_*a*,*i*_=*r*_*a*_ if *a*≠0, and *r*_*a*,0_=1, and similarly for SNP *b*. For each epistasis model we consider all the combination of MAF, LD, main effect and interaction parameter

For step three *n*_1_+*n*_0_ cases and controls are selected at random without replacement from the *m* individuals with probability *p*_*gwas*_=*n*_1_*p*_*k*_/(*mK*)+*n*_0_(1−*p*_*k*_)/(*m*−*mK*). Cases and controls are then affected using the backward sampling method waffect introduced by Perduca et al. [23]. This step is repeated for each disease scenario replicate. In the present work *n*_0_=*n*_1_=1000 and 500 replicates are generated per scenario. The number of replicates were chosen as a compromise between statistical accuracy and computation time, 2 months on a 30 Tesla K40 GPU cluster. The cohort size corresponds to a medium size GWAS and is sufficient to observe dominant-dominant epistasis effect with parameter *ρ*≈4 with a reasonable power. The relationship between the cohort size and the statistical power of each method for various scenarios is presented in the “Results” section.

### Methods benchmark

The following bivariate methods are selected for comparison: SHEsisEpi [26], fastepi [25], IndOR [13], DSS [14], and GBOOST [27]. All analyses are performed on Nvidia Tesla K40 graphic cards. We use the GPU implementation provided by their respective authors for GBOOST and DSS, and a GPU implementation in the GWISFI platform [31] for the other methods. The 2*χ*^2^ test [32] included in the GWISFI distribution is excluded from the analysis because the related score diverges for contingency tables with at least one empty cell. Numerical issues can occur as well for IndOR when inverting the covariance matrix *V*_*Φ*_ (see [13] for definition). This issue is treated by affecting a zero score to SNP pairs with non invertible *V*_*Φ*_. The information gain [33] and epiblaster [34] methods implemented in the GWISFI distribution require computing empirical *p*-value and are not included in the benchmark.

For each replication of each scenario, all 5 methods are applied to detect the SNP pair in epistasis in the resulting GWAS. Each method outputs a list of SNP pairs ranked by their respective epistasis score which were converted to *p*-value using the asymptotic score distribution corresponding to each method. For a given *p*-value threshold *p*_0_ the True Positive Rate (TPR) for a given method and scenario is defined at a SNP level as the probability that the causal pair (*a*,*b*) has an epistasis *p*-value *p*<*p*_0_, and the False Positive Rate (FPR) as the mean number of non-causal pairs with epistasis *p*-value *p*<*p*_0_ normalized by the number of SNP pairs. Grouping SNPs by LD blocks is a usual post-processing step in GWAS univariate analysis [35] and can be extended to the case of bivariate analysis. The LD blocks are defined a priori on the whole simulated population of size *m* using the plink options –blocks no-small-max-span –blocks-max-kb 500, which is based on the haplotype block definition of Haploview [36] suggested by Gabriel et al. [37]. SNPs that are not affected to a block via this procedure are affected to a single-SNP LD block. The whole set of SNPs map to 68819 LD blocks. The SNP pairs passing the epistasis *p*-value threshold are mapped to LD block pairs, with the causal LD block pair defined as the unique block pair (*A*,*B*) such that (*a*,*b*)∈(*A*,*B*) with (*a*,*b*) the causal SNP pair. TPR (resp. FPR) is defined at LD block level as the detection rate of the causal block pair (resp. the mean detection rate of the non causal block pairs).

The effect of the disease parameters on statistical power is evaluated by performing a canonical correlation analysis between (i) the power of the methods for each scenarios (normalized matrix *P*_*ij*_ for method *i* and scenarios *j*) and (ii) the value of the parameters for each scenarios (normalized matrix *V*_*kj*_ for parameter *k* and scenario *j*).

The effect of cohort size and MAF on statistical power is assessed in a separate set of simulations that consider only two SNPs. The disease penetrance *P*(*D*|*G*_*k*_) for each 9 genotypes *G*_*k*_∈{0,1,2}^2^ is computed as in step two of the previous three step simulation (Eq. 1). The genotypes of the *n*_0_ controls and *n*_1_ cases are simulated using Bayes formula *P*(*G*_*k*_|*D*)=*P*(*D*|*G*_*k*_)*P*(*G*_*k*_)/*K*, with the genotype frequency *P*(*G*_*k*_) controlled by *f*_*a*_=*f*_*b*_=*f* and *r*^2^. For the simulations with varying cohort size we consider *f*=0.3, *r*^2^∈{0,0.2}, dominant-dominant epistasis models (model *M*_1_ in Table 1) with main effect (*r*_*a*_,*r*_*b*_)∈{(1,1),(1.5,1),(1.5,1.5)}, and GWAS with *n*_0_=*n*_1_=*n*, *n*∈{500,1000,2500,5000,10000}. For the simulation with varying MAF we consider *n*=1000. For a given scenario and epistasis parameter *ρ* we compute the statistical power in 1000 replicates with a *p*-value threshold *p*=0.05/(1.25×10^10^), corresponding to a threshold *p*=0.05 with a Bonferroni correction for a bivariate analysis of a GWAS with 5×10^5^ SNPs. For each scenario and method the epistasis parameter *ρ* giving a power 0.8 is identified using Brent’s algorithm [38]. For this set of simulations GBOOST and DSS were reimplemented in Python using the information provided by the authors in [16, 39] respectively for computational performances reasons.

### Bivariate analysis of the WTCCC T2D

We applied each method on the WTCCC GWAS data on T2D [28], genotyped on the Affymetrix 5.0 platform. The standard Quality Control (QC) procedure suggested by the WTCCC is applied to the dataset except that SNPs with missing data are excluded because the bivariate methods implemented in the GWIS platform do not handle missing data. 363387 SNPs, 1953 cases and 2978 controls remain after QC. For each method interacting SNPs with *FDR*<0.05 are selected. Univariate association was performed using plink with the –fisher option. LD blocks are defined as in the simulations using the plink command –blocks no-small-max-span –blocks-max-kb 500 and SNPs are mapped to LD blocks in a n-1 relation. Each block is associated to a gene if the block region overlap with the gene region augmented by a 10kb margin. Consistently with the simulations two genes (A,B) are defined in epistasis if it exists two snps (a,b) detected in significant epistasis such that *a*∈*A* and *b*∈*B*).

## Results

### Type 1 error rate

**Fig. 1:**
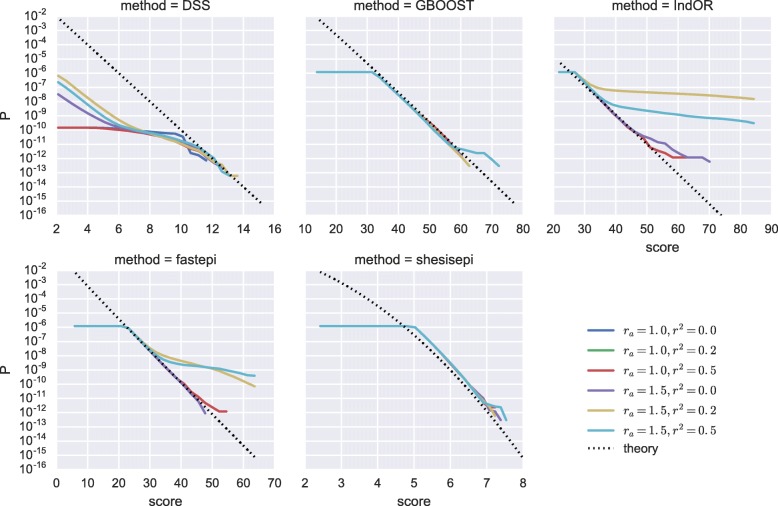
Score distribution in *M*_0_ simulations (no epistasis). Survival function of the scores (*SF*(score)=*P*(*S*>score)) output by each method for various *M*_0_ models (continuous line) and theoretical survival functions under *H*_0_ (dashed line). For IndOR, fastepi and SHEsisEpi only the top 10^4^ pairs are observed and the survival function therefore reach a plateau for *P*>10^4^/*n*_*pairs*_=1.19×10^−6^. For GBOOST a score threshold was set at 30 and therefore reach a plateau for lower scores. For DSS a score −*log*_10_(*flt*_*DSS*_) is returned by the software only for pairs passing a prefilter test as defined in [14], thus overestimating the *p*-value for small scores

**Fig. 2:**
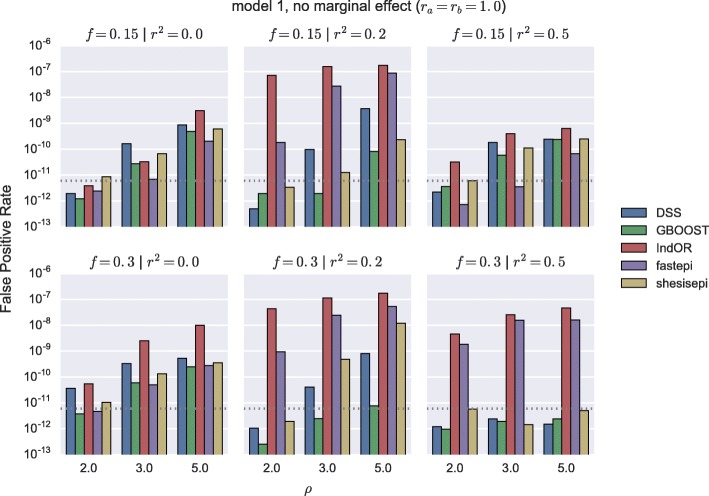
False positive rate in presence of epistasis without marginal effect. False Positive Rate with a *p*-value treshold after Bonferroni correction 0.05/*n*_*pairs*_=5.99×10^−12^ (dashed line). Model 1 with no marginal effect (*r*_*a*_=*r*_*b*_=1.0)

**Fig. 3:**
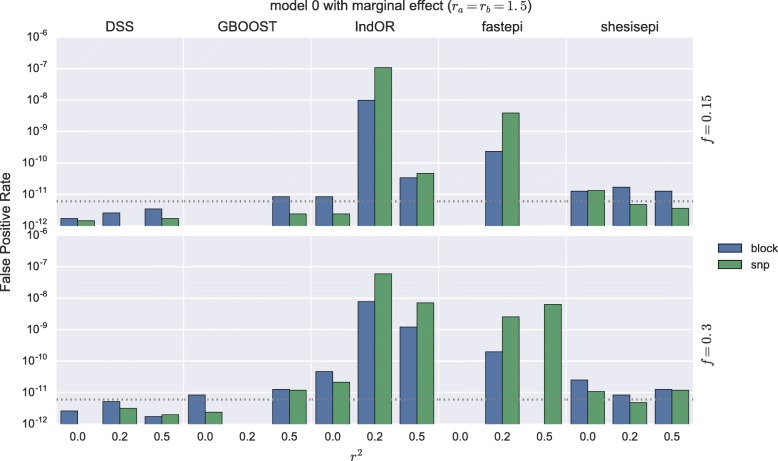
False positive rate at SNP and block level in absence of epistasis and with marginal effect

The score distributions of each method in several *M*_0_ simulations are represented in Fig. 1. In the absence of marginal effect the tail of the score distribution of all five methods is well described by their asymptotic distribution, even in presence of a realistic LD structure. We note that for high scores (> 50) the FPR of IndOR is underestimated by its asymptotic distribution. In the presence of a main effect on two SNPs in LD an important inflation of the far tail distribution is observed for IndOR and fastepi. The FPR for *M*_1_ simulations with no main effect, i.e. the ratio of non-causal pairs detected, is depicted in Fig. 2 for a *p*-value threshold of 0.05 after Bonferroni correction and present a similar behaviour: a good, slightly conservative, control of the FPR is observed for DSS, GBOOST and SHEsisEpi, while a two- to five-folds FPR increase is observed for IndOR and fastepi in presence of two SNPs in LD and in epistasis. Similar conclusions can be made in the *M*_2_, *M*_3_ and *M*_4_ models (Additional file 1: Figures). These findings suggest that grouping SNPs by LD blocks could improve the FPR control of IndOR and fastepi. The FPR at a SNP and LD block level are compared in Fig. 3 in the absence of epistasis and with main effect. In presence of SNPs in LD the FPR of IndOR and fastepi is only improved by one-fold at block level compared to the SNP level.

These observations indicate that analysis using IndOR and fastepi should focus on interactions between SNPs that are not in LD to ensure a good control of the false positive rate (under the definition of epistasis we assume in this work). Moreover caution should be taken when interpreting IndOR *p*-values for high scores. As mentioned in the “Methods” section, computing the IndOR score requires an inversion of the estimation of a variance-covariance matrix *V*_*Φ*_ (see [13] for definitions), which can be singular with a non zero probability for finite cohort sizes. In the authors R implementation and in the present work, this issue is treated by affecting zero to SNP pairs with non invertible *V*_*Φ*_. However, given the number of tests performed in exhaustive bivariate analysis of GWAS, such a situation occurs at an important rate among SNPs with high scores and a deviation from the asymptotic $\chi ^{2}_{4}$ distribution in the far tail can be expected. This situation highlights the importance of evaluating the performance of statistical methods on GWAS simulations of real size to identify finite size effects affecting method performances.

### Statistical power

**Fig. 4:**
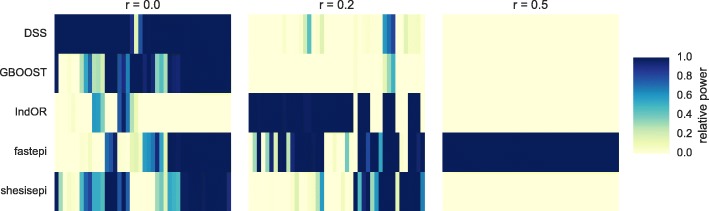
Methods relative power. Power of each method (rows) normalized by the highest power for each scenario (columns). scenarios in which no method achieved a power larger than 0.01 were excluded. Panels represent results for LD parameter *r*^2^=0, *r*^2^=0.2, and *r*^2^=0.5 from left to right

**Fig. 5:**
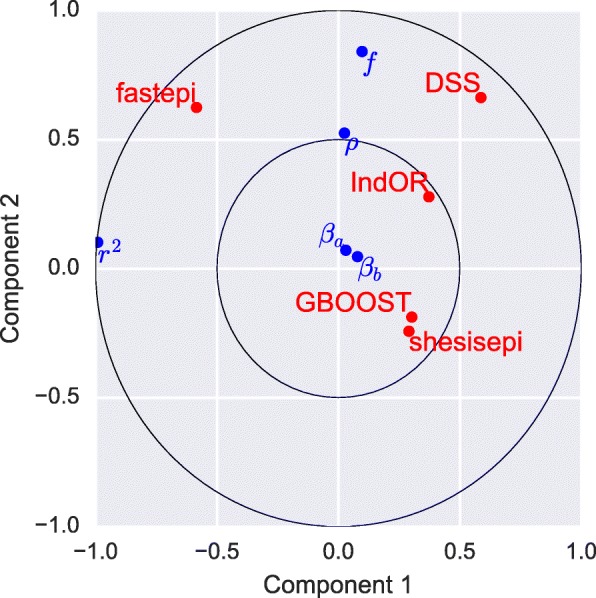
Canonical correlation analysis of methods power and disease parameter. Two first components of the canonical correlation analysis between the power of each method in all scenarios and the scenarios parameters: *ρ*, *r*^2^, *f*, *r*_*a*_ and *r*_*b*_

**Fig. 6:**
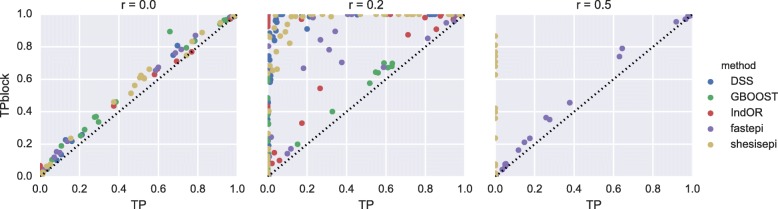
Power at block and SNP level. Each point represents for a given method and scenario the True Positive Rate (TP) at SNP level (x-axis) vs power at block level (y-axis). Panels represent results for LD parameter *r*^2^=0, *r*^2^=0.2, and *r*^2^=0.5 from left to right. The estimated power of DSS, GBOOST and IndOR for *r*^2^=0.5 is null at SNP and block level for all scenario and are not represented in the right panel

The statistical power of each method is evaluated with a Bonferroni corrected *p*-value threshold of 0.05 as in the previous section. The relative power of each method is reported in Fig. 4: fastepi is the most powerful method in 61 scenarios, IndOR in 45, DSS in 38, GBOOST in 5, and SHEsisEpi in 5. Among methods with a good FDR control DSS is the most powerful in most scenarios (46 vs 41 for SHEsisEpi). However fastepi was the only method with a non null measured power for scenarios with strong LD *r*^2^=0.5. Interestingly DSS is the most powerful method in almost all scenarios with no LD. GBOOST was the most powerful method in 5 scenarios with no LD, while none of the remaining method were the most powerful in any of the scenarios. The influence of the disease parameters on the methods power is illustrated through a canonical correlation analysis in Fig. 5, where we focus on the two first components. Component 1 is anti-correlated to *r*^2^, whereas component 2 is positively correlated to *f* and *ρ*. *β*_*a*_ and *β*_*b*_ are not correlated to components 1 or 2. The power of DSS and fastepi are similarly correlated to *f* and *ρ* (component 1) but the power of DSS is anticorrelated to *r*^2^, whereas the power of fastepi is positively correlated to *r*^2^ (as seen on Fig. 4). Conversely, the power of GBOOST and SHEsisEpi is not correlated to component 1 or component 2. The variations of power of GBOOST appear very similar to SHEsisEpi and the opposite to fastepi. This analysis also suggests as well that the variation of power of IndOR and DSS on one hand, and GBOOST, SHEsisEpi on the other are only weakly correlated. This observation is consistent across all models *M*_1_- *M*_4_ considered here (the separated canonical correlation analysis for each model are reported in Additional file 1). LD between the causal SNPs was the parameter most correlated to power variations, while the SNPs main effect coefficients *r*_*a*_, *r*_*b*_ were only weakly correlated. fastepi is the only method that shows a clear increase of power with LD. Figure 6 depicts the difference of power at SNP and LD block level for each method and scenario (one point per method-scenario). Analyses located on the first diagonal provided equal power at both the SNP and block level, while those in the top left have a gain in power when performing the analysis at SNP level. With no LD (*r*^2^=0) the difference of power at SNP and LD block level is limited for all methods. For intermediate LD (*r*^2^=0.2) a strong increase of power at LD block level was measured for all methods in almost all scenarios. For strong LD (*r*^2^=0.5) the estimated power of DSS, GBOOST and IndOR was null for all scenarios at SNP and LD block level, the estimated power of SHEsisEpi is null for all scenarios at SNP level and increases up to 0.86 at LD block level, whilst fastepi presented similar power at both level.

For studies focusing on interaction between SNPs that are not in LD our results indicate that DSS will be the most powerful method, while maintaining good control of the FPR. GBOOST appears as to be a good complementary solution to DSS.

### Impact of cohort size

Table 2 summarizes for cohort sizes ranging from *n*=*n*_0_=*n*_1_=500 to *n*=10,000 the smallest epistasis effects *ρ*_0.8_ detectable at a power of 0.8 for each method, for dominant-dominant epistasis. For *n*=500 no method can detect epistasis effect *ρ*<5 between common variants (*f*>0.3), while for *n*=5000 IndOR, DSS, GBOOST and SHEsisEpi can detect epistasis effect *ρ*<2. DSS is the most powerful method in all simulations, except for small cohort sizes (*n*≤1000), small MAF (*f*=0.15), and one SNP with no main effect (*r*_*a*_=1), were GBOOST is the most powerful. Results for all models and for LD values in {0.,0.2,0.5} are reported in the Additional file 1.

**Table 2:** Smallest epistasis effect *ρ* detectable with a power 0.8 for each method for various cohort size

	*f*=0.15					*f*=0.3					
Method	IndOR	DSS	fastepi	GBOOST	SHEsisEpi	IndOR	DSS	fastepi	GBOOST	SHEsisEpi	
*n*=*n*_0_=*n*_1_	*ρ*										
500	> 20	8.2	> 20	6.9	12.9	10.5	4.6	> 20	8.1	10.5	*r*_*a*_=1
1000	> 20	4.6	10.5	4.3	5.5	4.6	2.8	> 20	4.6	4.6	*r*_*b*_=1
2500	> 20	2.4	3.4	2.5	2.9	2.3	1.6	4.6	2.5	2.5	
5000	> 20	1.7	2.5	2.0	2.1	1.8	1.4	2.6	1.9	1.8	
10000	> 20	1.4	1.8	1.6	1.7	1.5	1.2	1.8	1.6	1.5	
500	> 20	8.7	> 20	6.9	15.2	> 20	4.6	> 20	8.7	15.2	*r*_*a*_=1
1000	> 20	4.7	12.9	4.0	5.8	4.6	3.0	> 20	4.6	4.7	*r*_*b*_=1.5
2500	> 20	2.5	3.7	2.5	2.8	2.4	1.6	6.3	2.6	2.5	
5000	> 20	1.8	2.4	1.9	2.0	1.8	1.4	2.8	1.9	1.9	
10000	> 20	1.4	1.9	1.6	1.7	1.5	1.2	2.0	1.6	1.5	
500	> 20	5.8	> 20	8.1	> 20	> 20	3.4	> 20	8.1	> 20	*r*_*a*_=1.5
1000	> 20	3.2	12.9	4.0	5.8	5.8	2.0	> 20	4.3	5.2	*r*_*b*_=1.5
2500	> 20	1.9	4.0	2.4	2.8	2.6	1.1	8.1	2.5	2.6	
5000	> 20	1.5	2.5	1.9	2.1	2.0	1.2	3.1	1.9	1.9	
10000	> 20	1.2	1.9	1.5	1.7	1.6	1.1	2.0	1.5	1.5	

Dominant-dominant model (*M*_1_ in Table 1), *f*_*a*_=*f*_*b*_=0.15 (left columns) and *f*_*a*_=*f*_*b*_=0.3 (right columns), *r*^2^=0, from top to bottom panel interaction between SNPs with no main effects (*r*_*a*_=*r*_*b*_=1), only one with main effects (*r*_*a*_=1,*r*_*b*_=1.5), and both with main effect (*r*_*a*_=*r*_*b*_=1.5)

### Impact of MAF

**Fig. 7:**
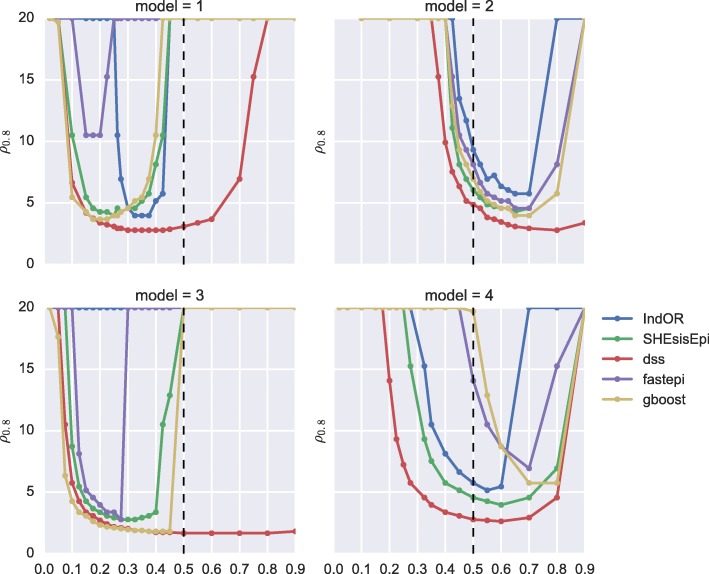
Influence of MAF on the smallest epistasis effect detectable. Smallest epistasis effect *ρ* detectable with a power 0.8 for each method depending on the MAF of causal SNPs (*f*_*a*_=*f*_*b*_=*f*). *n*_0_=*n*_1_=1000, and *β*_*a*_*β*_*b*_=1.0 (no main effect)

Figure 7 depicts the influence of MAF on the smallest epistasis effect *ρ*_0.8_ detectable with a power of 0.8. Results are given for each method, and for the 4 disease models *M*_1_ to *M*_4_. DSS is the most powerful method in all scenarios considered, except for *f*<0.2 in the dominant-dominant (*M*_1_) model and for *f*<0.3 in the multiplicative (*M*_3_) model, where GBOOST is slightly more powerful. The good performance of GBOOST in the multiplicative model can be explained by the simulated model corresponding to the full logistic regression model used in the likelihood ratio test underlying GBOOST. For models *M*_1_ and *M*3 all methods except DSS reach maximum power between *f*=0.15 and *f*=0.35 with a significant decrease in power in the vicinity of *f*=0.5. In *M*_1_ models epistasis effect *ρ*<5 can be detected by DSS for *f*>0.125, by GBOOST for 0.125<*f*<0.3, by SHEsisEpi for 0.175<*f*<0.25, and by IndOR for 0.3<*f*<0.4. Fastepi is not able to detect epistasis effects *ρ*<10 and reachs a maximum power at *f*=0.175.

### AUC comparison.

**Fig. 8:**
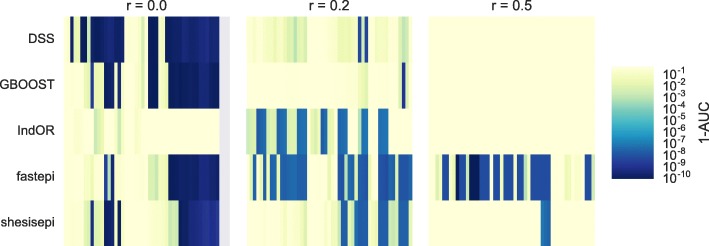
Area under the ROC curve. The global performance of each method is evaluated through the Area Under its ROC Curve (AUC). A piecewise linear approximation of the ROC curve is used to compute its AUC. Random classifiers area caracterized by *AUC*=0.5 and perfect classifiers by *AUC*=1. The AUC is represented for each method (rows) and each scenarios (columns), classified by their LD

To have a global view of how the methods perform we compute the Receiver Operating Characteristic (ROC) curves, defined by the true positive detection rate of the causal pair of SNPs vs the false positive rate for various *p*-value thresholds. The area under the ROC curve (AUC) is represented for each method and each scenario in Fig. 8. This approach directly evaluates the capacity of each method to separate causal SNPs pairs, independent of the method used to compute the *p*-value. As found previously DSS, presents the highest AUC for most scenarios with no LD. fastepi is the only method to efficiently identify causal SNP pairs with high LD values (*r*^2^=0.5). IndOR has the lowest AUC in all scenarios. Similar conclusions are found at a block level.

### Computational performance

Fastepi and SHEsisEpi are the two fastest methods (31s and 33s respectively), DSS has a intermediate speed (148s), and IndOR and GBOOST are the slowest methods (270s and 248s respectively). Table 3 reports measured and extrapolated computation time for 4 common SNP chips with up to 2.5M SNPs (Illumina Omni2.5-8) [40] and for cohort sizes up to 5 million (today GIANT is the largest GWAS identified and includes more than 300,000 patients [41]). As computation can be easily parallelized we can extrapolate that a giant GWAS with 5 million samples sequenced with an Omni2.5-8 Chip can be exhaustively analyzed in less than 20 days with DSS using a 30 Tesla K40 GPU cluster.

**Table 3:** Measured and extrapolated computation time of each method for various commercially available SNP chips and total number of samples in the GWAS

	Method	DSS	GBOOST	IndOR	fastepi	SHEsisEpi
snps	samples (k)					
0.6M (Axiom GW EU)	5	48.7 min*	1.8 h*	1.6 h*	13.5 min*	14.2 min*
	15	1.2 h*	5.3 h	4.8 h	32.5 min*	31 min*
	50	8.1 h	17.7 h	15.8 h	2.2 h	2.4 h
	300	2.0 days	4.4 days	4.0 days	13.5 h	14.2 h
	5000	1.1 months	2.5 months	2.2 months	9.4 days	9.9 days
0.7M (OmniExpress)	5	1.1 h	2.4 h	2.2 h	17 min*	19 min*
	15	3.3 h	7.2 h	6.5 h	55.1 min	58.0 min
	50	11.0 h	1.0 days	21.6 h	3.1 h	3.2 h
	300	2.8 days	6.0 days	5.4 days	18.4 h	19.3 h
	5000	1.5 months	3.4 months	3.0 months	12.7 days	13.4 days
1M (Omni1S-8)	5	2 h*	4.9 h	4.4 h	33 min*	35 min*
	15	6.8 h	14.8 h	13.2 h	1.9 h	2.0 h
	50	22.5 h	2.1 days	1.8 days	6.2 h	6.6 h
	300	5.6 days	12.3 days	11.0 days	1.6 days	1.6 days
	5000	3.1 months	6.8 months	6.1 months	26.0 days	27.4 days
2.5M (Omni2.5-8)	5	14.1 h	1.3 days	1.1 days	3.9 h	4.1 h
	15	1.8 days	3.8 days	3.4 days	11.7 h	12.3 h
	50	5.9 days	12.8 days	11.5 days	1.6 days	1.7 days
	300	1.2 months	2.6 months	2.3 months	9.8 days	10.3 days
	5000	1.6 years	3.5 years	3.1 years	5.4 months	5.7 months

Extrapolation based on one Nvidia Tesla K40 with the present implementation. Comutation time measured are indicated with a star

### Application to WTCCC T2D

Table 4 report the number of SNP pairs with *FDR*<0.05 for each method and the consensus between each two methods (off diagonal elements). IndOR detected 446 pairs in interactions but as observed above one has to consider that the current implementation might not allow for an accurate control of the false positive. GBOOST detected 230 pairs, DSS 119, fastepi 9, and SHEsisEpi none. The lowest number of SNPs detected by fastepi is coherent with the lowest power of the method observed in the simulations. While there is a relative large overlap between the pairs detected by GBOOST and IndOR, DSS detected a completely distinct set of pairs from the other methods.

**Table 4:** Number of SNP pairs with FDR<0.05 for each method (diagonal) and overlap between the pairs detected between two methods (off diagonal)

	GBOOST	IndOR	fastepi	DSS	SHEsisEpi
GBOOST	230	143	6	0	0
IndOR	143	446	2	0	0
fastepi	6	2	9	0	0
DSS	0	0	0	119	0
SHEsisEpi	0	0	0	0	0

Table 5 represents the overlap between the SNPs detected by epistasis tests and those detected by univariate association tests. The majority of SNPs detected in epistasis are not detected by univariate test (388 pairs over 654 have no detected main effect). IndOR is the only method to detect epistasis between SNPs having both main effects. We notice that in this situation our simulations indicate that the type 1 error rate might be incorrectly controlled by IndOR. None of the SNPs detected by DSS are detected by univariate test. Finally the majority of SNP pairs detected by gboost presents a main effect on one of the two SNPs.

**Table 5:** For each method number of SNP pairs (a,b) detected in interaction (FDR<0.05) such that (first line) both a and b are identified by univariate association test (FDR<0.05), (second line) one of a or b is identified, and (last line) neither a or b is identified

	IndOR	DSS	fastepi	gboost	SHEsisEpi
a and b	33	0	0	0	0
a xor b	189	0	2	174	0
none	224	119	7	56	0

These observations confirm that GBOOST and DSS detects different types of epistasis and could be combined for a potential increase in power.

**Fig. 9:**
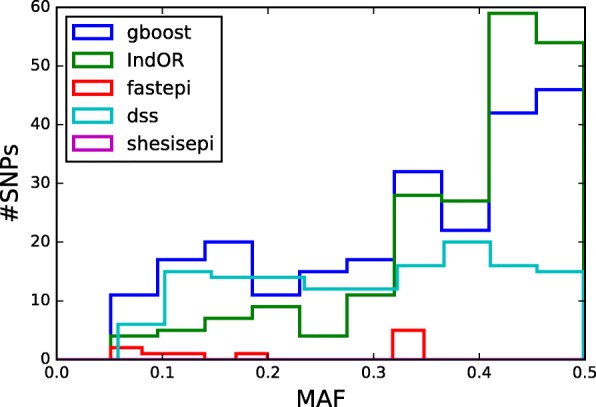
MAFs of SNPs detected in epistasis in the WTCCC GWAS on T2D. For each method MAF distribution of the SNPs in a pair detected in epistasis

**Fig. 10:**
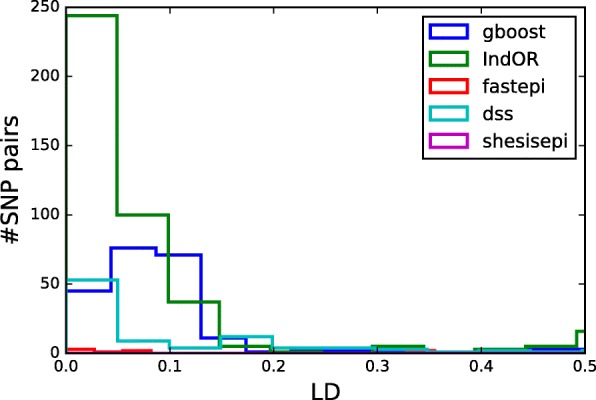
LD of SNP pairs detected in epistasis in the WTCCC GWAS on T2D. For each method LD distribution of the SNP pairs detected in epistasis

Figure 9 presents the distribution of MAF for the SNPs detected by each method. For DSS the distribution of MAF is uniform between 0.1 and 0.5 which is coherent with the results of the simulations. The pairs detected by gboost and IndOR are present a larger proportion of common SNPs with MAF greater than 0.3. This observation is coherent with the results of the simulations in non-dominant models (*M*_2_- *M*_4_). Figure 10 depicts the distribution of LD of the SNP pairs detected by each method. Most SNP pairs are in linkage equilibrium of in weak LD (*r*^2^=0.05 to 0.01). GBOOST is the only method to detect a larger proportion of SNP pairs in weak LD.

**Fig. 11:**
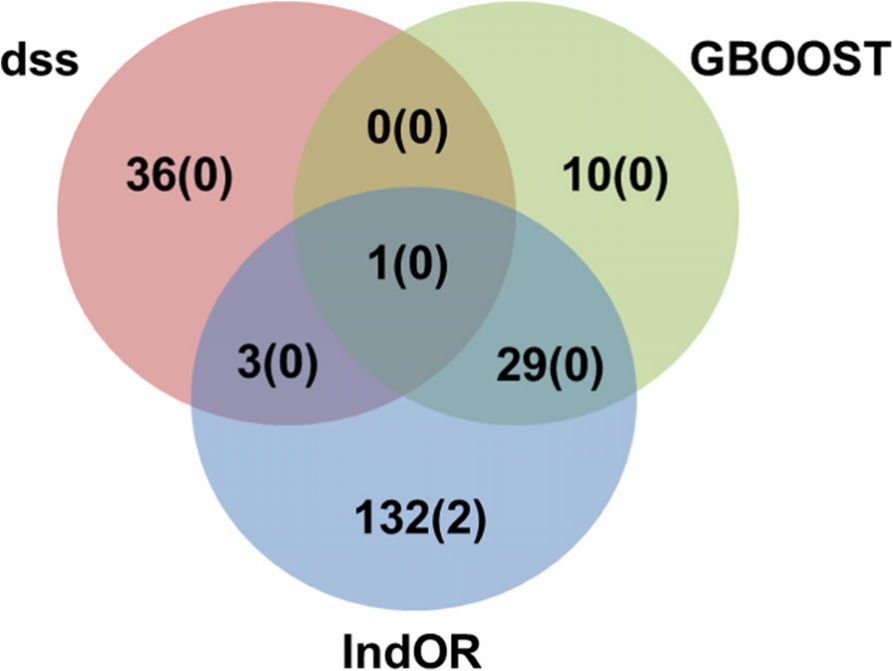
Venn diagramm of the genes detected by each method (the number of genes that were detect in univariate analysis is indicated in parenthesis)

The type of genomic regions including SNPs with interactions are reported in Table 6. IndOR is the only method to detect more interaction between protein coding regions. This observation might indicates that IndOR tends to detect interactions corresponding to a biological definition of epistasis as claimed by its authors [13]. GBOOST and DSS detect mostly epistasis between SNPs in intergenic regions. Among the 211 genes detected by the three methods DSS, GBOOST and IndOR only 2 are detected by univariate tests (Fig. 11).The Genes detected by DSS are distinct from those detected by other methods (10% of overlap only), which is consistent with the observation at SNP level. Only two genes are detected by univariate association tests. Interestingly each method detects distinct gene-gene interactions (see the epistasis networks in the Additional file 1).

**Table 6:** Number of SNP pair (a,b) identified on each type of locus pair for each method

		fastepi	GBOOST	IndOR	DSS	SHEsisEpi
Locus type a	Locus type b					
RNA, long non-coding	RNA, long non-coding	0	0	0	2	0
	RNA, micro	0	1	0	0	0
	gene with protein product	0	2	6	0	0
RNA, micro	RNA, micro	0	0	0	1	0
	gene with protein product	0	0	0	2	0
	intergenic	0	4	4	2	0
gene with protein product	RNA, long non-coding	0	0	14	2	0
	RNA, micro	0	0	1	1	0
	gene with protein product	2	22	148	17	0
	intergenic	0	23	104	6	0
	pseudogene	0	1	1	0	0
intergenic	RNA, long non-coding	0	5	4	0	0
	RNA, micro	0	3	2	0	0
	gene with protein product	2	69	74	10	0
	intergenic	5	105	89	68	0
pseudogene	gene with protein product	0	1	0	0	0
	pseudogene	0	1	0	0	0

## Discussion

Ten years after the first large scale GWAS from the Welcome Trust Case Control Consortium (WTCCC) [28] many results are available about the influence of single variants on various phenotypes. More than 35000 unique SNP-trait associations with *p*-values < 10^−8^ are reported in GWAS catalog [42]. Meta-analysis and replication studies [43] have identified and validated many susceptibility loci, for instance in type 2 diabetes [44], Alzheimer’s disease [45], cardiometabolic diseases [46], and ovarian cancer [47]. A large panel of tools are available to interpret the association at a biological level, these include fine mapping of GWAS signal, genome annotation, SNP prioritization tools, and gene set analysis tools such as DEPICT [41, 48]. Conversely results regarding genetic interactions, expected to explain an important proportion of the heritability of complex diseases [7], are far less established. In particular, accurate control of the power and type 1 error rate of the statistical tests of interaction in real GWAS has still to be improved. Indeed, recent studies on the WTCCC datasets have identified that significant differences exist in terms of stability and results overlap between epistasis detection methods [14, 49].

The objective of this work was to evaluate the performances of epistasis detection methods in quasi real GWAS conditions, where true causal interactions are known. We introduced a new GWAS simulation pipeline that combines the advantages of previous simulations approaches: generation of a large set of genetic samples with realistic LD [22], and simulation of a realistic GWAS cohort that can take into account disease models with epistasis between SNPs in LD using backward sampling [23]. We evaluated five epistasis detection methods using this pipeline: SHEsisEpi [26], fastepi [25], IndOR [13], DSS [14], and GBOOST [27]. In total 234 different disease models were considered when generating GWAS, with 1000 cases and controls and 129,238 SNPs reproducing the LD structure of the EUR group of the 1000 Genomes Project [29].

Our results indicate that using the asymptotic *χ*^2^ distribution to compute the *p*-value of IndOR and fastepi does not enable an accurate control of the false positive rate in presence of causal SNPs in LD. With respect to IndOR the first reason for the apparent inaccurate control comes from the definition of epistasis. IndOR relies on a definition of epistasis based on Odds ratio independence [13] different from the statistical definition assumed in this paper. Only biological investigation can validate whether more true biological interactions are detected using one definition or the other. The second reason is numerical. Fixing an arbitrary score to SNP pairs with a singular *V*_*ϕ*_ matrix is expected to modify the score distribution. For GBOOST, DSS and SHEsisEpi the *p*-value computed from the asymptotic *χ*^2^ distribution allows an acceptable control of the false positive rate, with Bonferroni found to be slightly conservative, due to the correlation between SNPs. When implementing epistasis detection methods it should be noted that contingency tables with empty cells occurs at an important rate: assuming independent SNPs with uniform MAF distribution between 0.05 and 0.5 the probability of having a bivariate contingency table with no double recessive genotype in a GWAS of 1000 samples is $\int _{0.05}^{0.5}\left (1-f^{4}\right)^{1000}/0.45 = 0.25$. Using a Axiom GW EU chip more than 10^10^ such contingency tables would be expected, for which the *χ*^2^ approximation can be invalid, and numerical issues can appear as in the case of the 2*χ*^2^ test [32] implemented in the GWISFI distribution.

DSS is the most powerful method in most scenarios with no LD between the causal SNPs, with GBOOST being a good complementary solution to DSS with close performances in terms of power. In the presence of LD between the causal SNPs, IndOR is the most powerful method in most scenarios. However, given the more important false positive rate of this method the false discovery rate might be higher. Given that the power of DSS and GBOOST are influenced differently by the disease model parameter, a combination approach combining both scores might improve the overall power. This strategy is to be supported by the results on the WTCCC data on T2D were DSS and GBOOST detected distinct SNPs and genes in epistasis.

DSS also has the largest AUC for almost all scenarios, which confirms the good performance of the method. IndOR had the lowest AUC in all scenarios, which can be explained in part, by the different epistasis definition underlying the method.

Similar results are obtained when grouping SNPs by LD blocks, with only a limited increase of power for SNP pairs that are not in LD, and an important increase of power for SNP pairs in LD.

We show that strong dominant-dominant (*M*_1_) and multiplicative (*M*_3_) epistasis effects expected to lie in the range 2<*ρ*<5 can be detected with sufficient power by all methods except fastepi in relatively small cohort size of 2000 cases and controls. However we show that such interaction will only be detected in a narrow MAF window, except for DSS which is the only method able to detect interacting SNPs with MAF ranging from 0.1 to close to 0.5. Detecting weak epistasis effects *ρ*<2 will require GWAS with more than 10,000 cases and controls, which may become available in the coming years.

We conclude that computation time is no longer a limiting issue to perform exhaustive bivariate interaction analysis of a large GWAS. Our results suggest that SNPs with no or weak LD (*r*^2^<0.2) should be analyzed with DSS complemented with GBOOST in order to achieve the best statistical performances. The analysis of SNPs in LD is more complicated, because fastepi is the best method identified in terms of AUC but does not allow for a good control of the FPR and also because most SNPs in LD are located in the a same functional DNA region. The biological interpretation of intra-region interaction can be more complicated than for intergenic interactions. A possible strategy could be, therefore, to focus only on SNP pairs with no LD which would give valuable information on the biology underlying the disease. If the missing heritability in GWAS can be explained by epistasis between common variants, the existing methods will be powerful enough to detect them in GWAS with as few as tens of thousands of cases plus controls, as is likely to be available in the coming years.

In terms of disease understanding and new therapeutic strategies, the most notable impact of these approaches might not be the additional heritability identified, but the insight into disease network biology. While univariate GWAS analysis can identify isolated candidate genes, epistasis methods additionally identify interaction networks between risk genes. Complex diseases such as Diabetes or NASH results from the dysregulation of complex genetic and metabolic networks. For at least a decade it has been argued that disease complexity and redundancy in biological pathways might be responsible for the limited effect of many monotherapies [50]. The recent success in cancer, HIV [51], and cardiology [52] multitarget therapies appears to confirm this idea, creating a high interest in multitarget discovery in other therapeutic area, which might be supported by epistasis analysis of GWAS.

## Conclusion

This study confirms that computation time is no longer a limiting factor for performing an exhaustive search of epistasis in large GWAS. For this task, using DSS on SNP pairs with limited LD seems to be a good strategy to achieve the best statistical performance. A combination approach using both DSS and GBOOST is supported by the simulation results and the analysis of the WTCCC dataset demonstrated that this approach can detect distinct genes in epistasis. Finally, weak epistasis between common variants will be detectable with existing methods when GWAS of a few tens of thousands cases and controls are available. The major remaining challenges are (i) the lack of unique and well defined epistasis definition at a biological level, and (ii) the relatively limited amount of tools for translating statistical interactions at a SNP level into biological mechanisms. Several tools have recently emerged to fill this last gap. For instance Emily [18] and Stanislas et al. [19] have developed two methods for epistasis analysis at a gene level, Ma et al. [20], an epistasis analysis at a gene level for continuous phenotypes, and Lin et al. [53], introduced an epistasis test in meta-analysis. The present work could therefore be expanded to evaluate the performance of these new tools in a realistic framework. Other influencing factor should also be evaluated such as chip coverage and imputation, or the effect of population structure.

## Additional file

Additional file 1 Simulation results on all scenarios and epistasis networks detected in the T2D GWAS. (PDF 331 kb)
