# Supplementary material for: Performance of epistasis detection methods in semi-simulated GWAS
Source: BMC Bioinformatics. 2018 Jun 18;19:231. doi: 10.1186/s12859-018-2229-8 (PMC6006572; doi:10.1186/s12859-018-2229-8)
Supplement: Supplementary file 1 — Simulation results on all scenarios and epistasis networks detected in the T2D GWAS. (PDF 331 kb) [file 12859_2018_2229_MOESM1_ESM.pdf]

Performance of epistasis detection methods in semi-simulated GWAS
Supplementary Materials

Clément Chatelain, Guillermo Durand, Vincent Thuillier, and Franck Augé

1 Canonical correlation analysis of method power versus disease scenario parameters

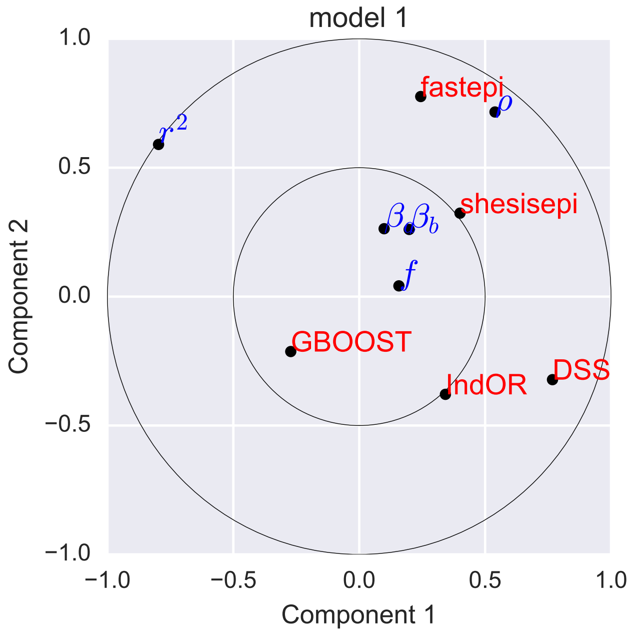

Figure 1: Canonical Correlation Analysis of Methods power and Disease parameter. Model M_1. Two first components of the canonical correlation analysis between the power of each method in all scenarios and the scenario parameters: ρ, r^2, f, r_a and r_b.

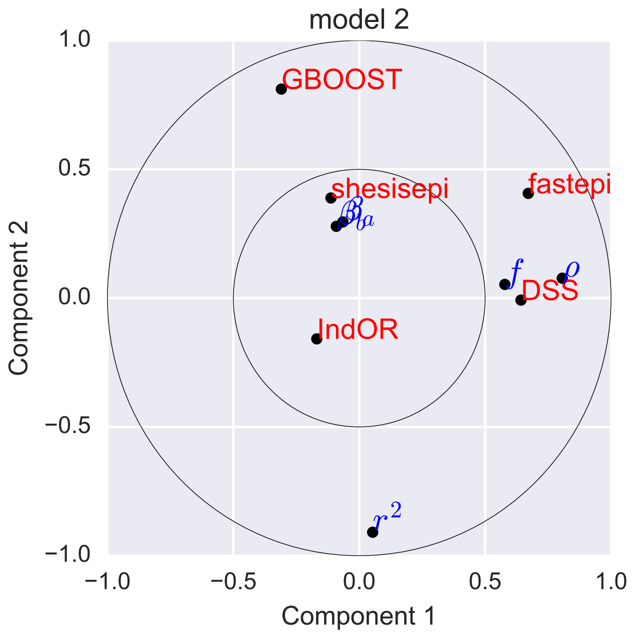

Figure 2: Canonical Correlation Analysis of Methods power and Disease parameter. Model M_2. Two first components of the canonical correlation analysis between the power of each method in all scenarios and the scenario parameters: ρ, r^2, f, r_a and r_b.

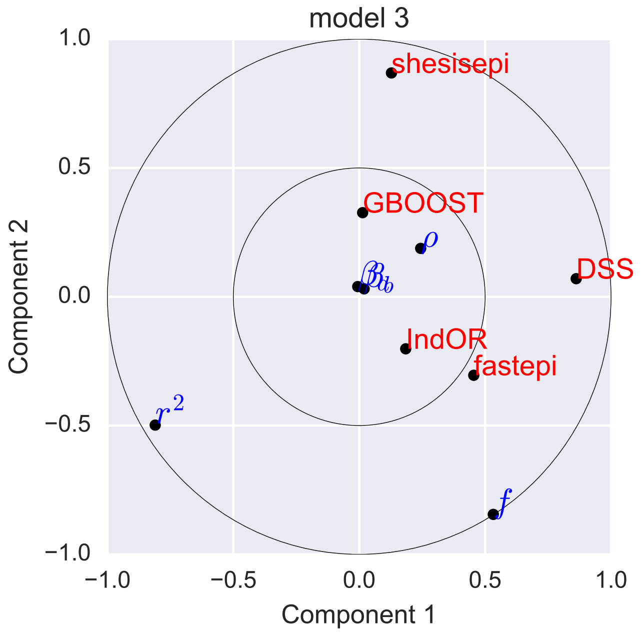

Figure 3: Canonical Correlation Analysis of Methods power and Disease parameter. Model M_3. Two first components of the canonical correlation analysis between the power of each method in all scenarios and the scenario parameters: ρ, r^2, f, r_a and r_b.

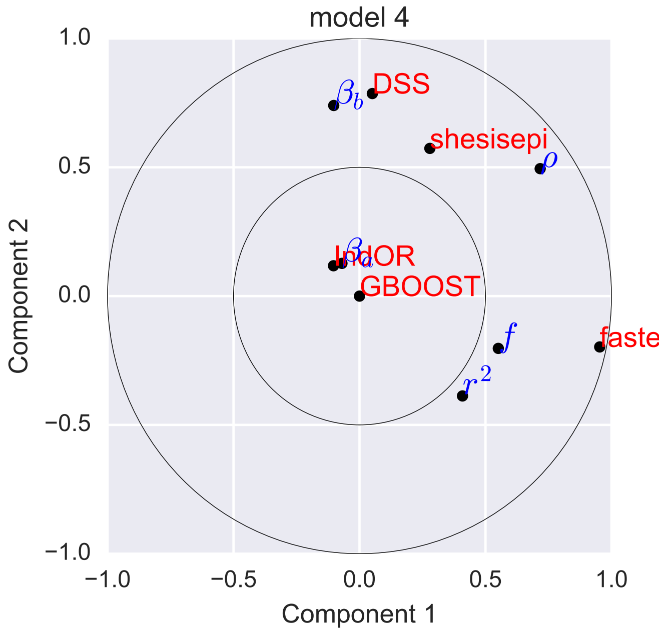

Figure 4: Canonical Correlation Analysis of Methods power and Disease parameter. Model M_4. Two first components of the canonical correlation analysis between the power of each method in all scenarios and the scenario parameters: ρ, r^2, f, r_a and r_b.

2 Influence of cohort size

2.1 model M_1: dominant-dominant

model M_1 : dominant-dominant with no main effect

Table 1: Smallest epistasis effect ρ detectable with a power 0.8 for each method for various cohort size. Model M_1 (dominant-dominant), and r_a = 1.0, r_b = 1.0 (no main effect).

		ρ									
	f	0.15					0.30				
	method	IndOR	SHEsisEpi	dss	fastepi	gboost	IndOR	SHEsisEpi	dss	fastepi	gboost
r^2	$n = n_0 = n_1$										
0.0	500	>20	12.9	8.2	>20	6.9	10.5	10.5	4.6	>20	8.1
	1000	>20	5.5	4.6	10.5	4.3	4.6	4.6	2.8	>20	4.6
	2500	>20	2.9	>20	3.4	2.5	2.3	2.5	10.8	4.6	2.5
	5000	>20	2.1	>20	2.5	2.0	1.8	1.8	>20	2.6	1.9
	10000	>20	1.7	>20	1.8	1.6	1.5	1.5	>20	1.8	1.6
0.2	500	>20	17.6	8.1	>20	>20	3.1	>20	6.2	>20	>20
	1000	4.4	6.0	4.3	>20	9.3	2.3	5.8	3.4	>20	>20
	2500	2.8	3.1	>20	>20	4.6	1.7	2.8	1.1	>20	>20
	5000	2.2	2.2	>20	>20	3.1	1.4	2.0	>20	14.7	>20
	10000	1.8	1.7	>20	>20	2.2	1.3	1.7	>20	9.6	>20
0.5	500	>20	>20	18.8	>20	>20	3.1	>20	13.5	>20	>20
	1000	2.7	15.2	6.3	>20	>20	2.2	18.8	5.2	10.5	18.8
	2500	2.0	4.6	>20	6.9	>20	1.7	4.1	1.1	4.6	8.1
	5000	1.7	2.8	>20	4.1	>20	1.4	2.4	>20	3.1	4.9
	10000	1.4	2.0	>20	3.0	12.9	1.2	1.8	>20	2.3	3.2

model M_1 : dominant-dominant with main effect on SNP a only

Table 2: Smallest epistasis effect ρ detectable with a power 0.8 for each method for various cohort size. Model M_1 (dominant-dominant), and r_a = 1.0, r_b = 1.5 (main effect on SNP a only).

		ρ									
	f	0.15					0.30				
	method	IndOR	SHEsisEpi	dss	fastepi	gboost	IndOR	SHEsisEpi	dss	fastepi	gboost
r^2	$n = n_0 = n_1$										
0.0	500	>20	15.2	8.7	>20	6.9	>20	15.2	4.6	>20	8.7
	1000	>20	5.8	4.7	12.9	4.0	4.6	4.7	3.0	>20	4.6
	2500	>20	2.8	>20	3.7	2.5	2.4	2.5	1.6	6.3	2.6
	5000	>20	2.0	>20	2.4	1.9	1.8	1.9	>20	2.8	1.9
	10000	>20	1.7	>20	1.9	1.6	1.5	1.5	>20	2.0	1.6
0.2	500	>20	18.8	8.1	>20	>20	2.2	>20	5.8	>20	>20
	1000	2.7	5.8	4.6	>20	9.3	1.7	8.1	3.4	>20	>20
	2500	1.7	3.1	>20	>20	4.6	1.2	2.8	1.6	>20	>20
	5000	1.2	2.2	>20	>20	3.4	1.1	2.0	>20	8.7	>20
	10000	1.1	1.7	>20	>20	2.5	1.1	1.7	>20	5.2	>20
0.5	500	>20	>20	15.2	>20	>20	2.0	>20	15.2	>20	>20
	1000	1.7	15.2	5.8	12.9	>20	1.5	17.6	5.2	6.3	11.7
	2500	1.2	4.6	>20	3.7	>20	1.1	3.7	1.1	2.6	4.6
	5000	1.1	2.8	>20	2.2	>20	1.1	2.3	>20	1.7	2.5
	10000	1.1	2.0	>20	1.5	12.9	1.1	1.7	>20	1.2	1.6

model M_1 : dominant-dominant with main effect on both SNPs

Table 3: Smallest epistasis effect ρ detectable with a power 0.8 for each method for various cohort size. Model M_1 (dominant-dominant), and r_a = 1.5, r_b = 1.5 (main effect on both SNPs).

		ρ									
	f	0.15					0.30				
	method	IndOR	SHEsisEpi	dss	fastepi	gboost	IndOR	SHEsisEpi	dss	fastepi	gboost
r^2	$n = n_0 = n_1$										
0.0	500	>20	>20	5.8	>20	8.1	>20	>20	3.4	>20	8.1
	1000	>20	5.8	3.2	12.9	4.0	5.8	5.2	2.0	>20	4.3
	2500	>20	2.8	>20	4.0	2.4	2.6	2.6	1.1	8.1	2.5
	5000	>20	2.1	>20	2.5	1.9	2.0	1.9	>20	3.1	1.9
	10000	>20	1.7	>20	1.9	1.5	1.6	1.5	>20	2.0	1.5
0.2	500	>20	>20	5.8	>20	>20	1.7	>20	4.6	>20	>20
	1000	1.6	6.3	3.2	>20	9.3	1.2	10.5	2.3	>20	>20
	2500	1.1	3.2	>20	>20	4.6	1.1	3.1	1.3	12.9	>20
	5000	1.1	2.2	>20	>20	3.4	1.1	2.0	>20	4.7	>20
	10000	1.1	1.7	>20	>20	2.6	1.1	1.7	>20	2.2	>20
0.5	500	>20	>20	12.9	>20	>20	1.4	>20	10.5	15.2	>20
	1000	1.1	17.6	4.6	6.9	>20	1.1	15.2	3.7	4.0	6.9
	2500	1.1	4.6	>20	2.0	>20	1.1	3.1	1.1	1.5	2.0
	5000	1.1	2.6	>20	1.1	>20	1.1	1.8	>20	1.1	1.1
	10000	1.1	1.9	>20	1.1	12.9	1.1	1.2	>20	1.1	1.1

2.2 model M_2: recessive-recessive

model M_2 : recessive-recessive with no main effect

Table 4: Smallest epistasis effect ρ detectable with a power 0.8 for each method for various cohort size. Model M_2 (recessive-recessive), and r_a = 1.0, r_b = 1.0 (no main effect).

	ρ										
	f	0.15					0.30				
	method	IndOR	SHEsisEpi	dss	fastepi	gboost	IndOR	SHEsisEpi	dss	fastepi	gboost
r^2	$n = n_0 = n_1$										
0.0	500	>20	>20	>20	>20	>20	>20	>20	>20	>20	>20
	1000	>20	>20	>20	>20	>20	>20	>20	>20	>20	>20
	2500	>20	>20	>20	>20	>20	12.9	>20	1.1	>20	>20
	5000	>20	>20	>20	>20	>20	5.8	6.3	>20	14.1	11.7
	10000	>20	>20	>20	>20	>20	3.4	3.4	>20	6.8	5.5
0.2	500	>20	>20	>20	>20	>20	>20	>20	>20	>20	>20
	1000	>20	>20	>20	>20	>20	11.1	>20	13.2	11.7	9.3
	2500	>20	>20	>20	>20	>20	4.6	5.2	1.1	5.2	4.0
	5000	15.2	18.8	>20	15.2	8.7	2.9	3.1	>20	3.1	2.6
	10000	6.0	5.8	>20	6.3	4.6	2.2	2.3	>20	2.3	2.0
0.5	500	>20	>20	>20	>20	>20	8.1	>20	>20	15.2	10.5
	1000	>20	>20	>20	>20	>20	4.6	14.1	18.8	6.3	5.2
	2500	7.5	>20	>20	15.2	6.9	2.7	4.6	>20	3.4	2.8
	5000	4.1	9.3	>20	6.3	4.0	2.0	3.1	>20	2.5	2.1
	10000	2.8	4.4	>20	4.0	2.6	1.7	2.1	>20	2.0	1.7

model M_2 : recessive-recessive with main effect on SNP a only

Table 5: Smallest epistasis effect ρ detectable with a power 0.8 for each method for various cohort size. Model M_2 (recessive-recessive), and r_a = 1.0, r_b = 1.5 (main effect on SNP a only).

		ρ									
	f	0.15					0.30				
	method	IndOR	SHEsisEpi	dss	fastepi	gboost	IndOR	SHEsisEpi	dss	fastepi	gboost
r^2	$n = n_0 = n_1$										
0.0	500	>20	>20	>20	>20	>20	>20	>20	>20	>20	>20
	1000	>20	>20	>20	>20	>20	>20	>20	>20	>20	>20
	2500	>20	>20	>20	>20	>20	17.6	>20	1.1	>20	>20
	5000	>20	>20	>20	>20	>20	6.9	6.3	>20	18.8	17.6
	10000	>20	>20	>20	>20	>20	3.7	3.4	>20	7.2	6.3
0.2	500	>20	>20	>20	>20	>20	17.6	>20	>20	>20	>20
	1000	>20	>20	>20	>20	>20	5.2	15.2	12.9	19.4	15.2
	2500	10.5	>20	>20	>20	>20	2.0	4.1	1.1	6.3	5.8
	5000	2.8	15.2	>20	>20	>20	1.1	2.5	>20	4.3	3.7
	10000	1.1	5.2	>20	17.6	8.1	1.1	1.8	>20	3.2	2.8
0.5	500	>20	>20	>20	>20	>20	5.8	>20	>20	>20	15.2
	1000	15.2	>20	>20	>20	>20	3.1	8.7	17.6	10.5	7.8
	2500	2.8	>20	>20	>20	15.2	1.6	3.3	>20	5.2	4.1
	5000	1.1	5.8	>20	>20	6.3	1.1	2.0	>20	3.8	3.0
	10000	1.1	3.1	>20	9.3	4.3	1.1	1.5	>20	3.0	2.4

model M_2 : recessive-recessive with main effect on both SNPs

Table 6: Smallest epistasis effect ρ detectable with a power 0.8 for each method for various cohort size. Model M_2 (recessive-recessive), and r_a = 1.5, r_b = 1.5 (main effect on both SNPs).

	ρ										
	f	0.15					0.30				
	method	IndOR	SHEsisEpi	dss	fastepi	gboost	IndOR	SHEsisEpi	dss	fastepi	gboost
r^2	$n = n_0 = n_1$										
0.0	500	>20	>20	>20	>20	>20	>20	>20	>20	>20	>20
	1000	>20	>20	>20	>20	>20	>20	>20	>20	>20	>20
	2500	>20	>20	>20	>20	>20	>20	>20	1.1	>20	>20
	5000	>20	>20	>20	>20	>20	9.3	6.9	>20	>20	>20
	10000	>20	>20	>20	>20	>20	4.3	3.5	>20	7.8	7.5
0.2	500	>20	>20	>20	>20	>20	6.9	>20	>20	>20	>20
	1000	>20	>20	>20	>20	>20	2.0	12.9	16.4	>20	>20
	2500	1.1	>20	>20	>20	>20	1.1	3.4	1.1	9.9	8.7
	5000	1.1	15.2	>20	>20	>20	1.1	2.0	>20	6.0	5.2
	10000	1.1	4.0	>20	>20	>20	1.1	1.4	>20	4.6	4.0
0.5	500	>20	>20	>20	>20	>20	3.2	>20	>20	>20	>20
	1000	2.2	>20	>20	>20	>20	1.1	6.0	18.8	>20	12.9
	2500	1.1	12.9	>20	>20	>20	1.1	2.5	>20	8.1	6.3
	5000	1.1	4.0	>20	>20	15.2	1.1	1.6	>20	5.9	4.4
	10000	1.1	2.2	>20	>20	7.2	1.1	1.1	>20	4.6	3.5

2.3 model M_3: multiplicative

model M_3 : multiplicative with no main effect

Table 7: Smallest epistasis effect ρ detectable with a power 0.8 for each method for various cohort size. Model M_3 (multiplicative), and r_a = 1.0, r_b = 1.0 (no main effect).

	ρ										
	f	0.15					0.30				
	method	IndOR	SHEsisEpi	dss	fastepi	gboost	IndOR	SHEsisEpi	dss	fastepi	gboost
r^2	$n = n_0 = n_1$										
0.0	500	>20	10.5	6.4	>20	4.6	>20	5.2	2.9	>20	2.8
	1000	>20	4.3	3.3	5.2	3.1	>20	2.8	2.0	>20	2.0
	2500	>20	2.4	>20	2.3	1.9	>20	1.8	>20	1.7	1.5
	5000	>20	1.9	>20	1.8	1.6	>20	1.5	>20	1.4	1.4
	10000	>20	1.5	>20	1.5	1.4	>20	1.4	>20	1.3	1.2
0.2	500	>20	12.9	5.8	>20	5.8	>20	>20	3.4	>20	3.4
	1000	>20	4.6	3.2	>20	2.8	>20	3.1	2.5	>20	2.0
	2500	>20	2.4	>20	>20	1.8	>20	1.9	>20	>20	1.5
	5000	>20	1.8	>20	>20	1.5	>20	1.5	>20	>20	1.4
	10000	>20	1.5	>20	>20	1.4	>20	1.4	>20	>20	1.2
0.5	500	>20	>20	12.9	>20	17.6	>20	>20	5.8	>20	>20
	1000	>20	10.5	4.7	>20	3.7	>20	>20	4.0	>20	2.0
	2500	>20	3.4	>20	>20	1.8	>20	1.7	>20	>20	1.5
	5000	>20	2.0	>20	12.3	1.5	>20	1.5	>20	>20	1.4
	10000	>20	1.7	>20	9.2	1.4	>20	1.4	>20	>20	1.2

model M_3 : multiplicative with main effect on SNP a only

Table 8: Smallest epistasis effect ρ detectable with a power 0.8 for each method for various cohort size. Model M_3 (multiplicative), and r_a = 1.0, r_b = 1.5 (main effect on SNP a only).

		ρ									
	f	0.15					0.30				
	method	IndOR	SHEsisEpi	dss	fastepi	gboost	IndOR	SHEsisEpi	dss	fastepi	gboost
r^2	$n = n_0 = n_1$										
0.0	500	>20	12.9	6.9	>20	5.8	>20	8.1	2.8	>20	2.9
	1000	>20	4.6	3.8	6.9	3.1	>20	3.1	2.2	>20	2.0
	2500	>20	2.4	>20	2.4	2.0	>20	1.9	>20	1.8	1.5
	5000	>20	1.8	>20	1.8	1.6	>20	1.5	>20	1.5	1.4
	10000	>20	1.5	>20	1.5	1.4	>20	1.4	>20	1.4	1.2
0.2	500	>20	15.2	6.3	>20	6.9	>20	>20	3.2	>20	4.6
	1000	>20	4.6	3.4	>20	3.1	>20	4.0	2.2	>20	2.5
	2500	>20	2.5	>20	>20	2.0	>20	1.8	>20	>20	1.7
	5000	>20	1.8	>20	>20	1.7	>20	1.5	>20	>20	1.5
	10000	>20	1.5	>20	>20	1.4	>20	1.3	>20	>20	1.4
0.5	500	>20	>20	12.9	>20	>20	>20	>20	5.2	>20	>20
	1000	>20	11.7	4.6	>20	4.6	>20	>20	3.7	>20	2.5
	2500	>20	3.4	>20	12.9	2.2	>20	1.6	>20	>20	1.8
	5000	>20	1.9	>20	7.5	1.7	>20	1.4	>20	>20	1.5
	10000	>20	1.5	>20	5.6	1.5	>20	1.2	>20	>20	1.4

model M_3 : multiplicative with main effect on both SNPs

Table 9: Smallest epistasis effect ρ detectable with a power 0.8 for each method for various cohort size. Model M_3 (multiplicative), and r_a = 1.5, r_b = 1.5 (main effect on both SNPs).

	ρ										
	f	0.15					0.30				
	method	IndOR	SHEsisEpi	dss	fastepi	gboost	IndOR	SHEsisEpi	dss	fastepi	gboost
r^2	$n = n_0 = n_1$										
0.0	500	>20	17.6	4.9	>20	5.5	>20	>20	2.1	>20	3.1
	1000	>20	4.9	2.7	8.1	3.1	>20	3.1	1.6	>20	2.0
	2500	>20	2.5	>20	2.6	2.0	>20	1.9	>20	1.9	1.5
	5000	>20	1.8	>20	1.9	1.6	>20	1.5	>20	1.5	1.4
	10000	>20	1.5	>20	1.5	1.4	>20	1.4	>20	1.4	1.2
0.2	500	>20	15.2	4.6	>20	6.9	>20	>20	2.6	>20	>20
	1000	>20	4.6	2.6	>20	3.4	>20	>20	1.7	>20	2.8
	2500	>20	2.5	>20	>20	2.2	>20	1.7	>20	>20	1.9
	5000	>20	1.8	>20	>20	1.8	>20	1.4	>20	>20	1.7
	10000	>20	1.5	>20	>20	1.5	>20	1.2	>20	>20	1.5
0.5	500	>20	>20	9.3	>20	>20	>20	>20	4.6	>20	>20
	1000	>20	15.2	3.7	>20	5.2	>20	>20	2.3	>20	>20
	2500	>20	3.4	>20	8.1	2.8	>20	1.4	>20	>20	2.2
	5000	>20	1.7	>20	4.6	2.0	>20	1.2	>20	>20	1.8
	10000	>20	1.4	>20	3.1	1.7	>20	1.1	>20	17.0	1.7

2.4 model M_4: alternative

model M_4 : alternative with no main effect

Table 10: Smallest epistasis effect ρ detectable with a power 0.8 for each method for various cohort size. Model M_4 (alternative), and r_a = 1.0, r_b = 1.0 (no main effect).

	ρ										
	f	0.15					0.30				
	method	IndOR	SHEsisEpi	dss	fastepi	gboost	IndOR	SHEsisEpi	dss	fastepi	gboost
r^2	$n = n_0 = n_1$										
0.0	500	>20	>20	>20	>20	>20	>20	>20	9.3	>20	>20
	1000	>20	>20	>20	>20	>20	>20	11.7	4.9	>20	>20
	2500	>20	>20	>20	>20	>20	4.9	4.3	>20	>20	>20
	5000	>20	12.9	>20	>20	>20	3.1	2.8	>20	>20	>20
	10000	>20	5.2	>20	>20	>20	2.3	2.1	>20	>20	8.1
0.2	500	>20	>20	>20	>20	>20	6.6	>20	8.1	>20	>20
	1000	>20	>20	17.6	>20	>20	3.8	15.2	4.6	>20	>20
	2500	9.3	>20	>20	>20	10.5	2.4	5.8	>20	>20	8.1
	5000	4.3	15.2	>20	15.2	4.9	1.8	3.6	>20	>20	3.7
	10000	2.8	5.8	>20	5.5	3.1	1.5	2.6	>20	>20	2.3
0.5	500	>20	>20	>20	>20	>20	5.5	>20	12.9	>20	>20
	1000	>20	>20	>20	>20	>20	3.1	15.2	6.0	>20	9.3
	2500	4.0	>20	>20	>20	5.8	2.0	5.5	>20	10.5	3.7
	5000	2.6	12.9	>20	9.3	3.5	1.7	3.7	>20	4.0	2.5
	10000	2.0	6.3	>20	4.6	2.5	1.4	2.5	>20	2.5	1.9

model M_4 : alternative with main effect on SNP a only

Table 11: Smallest epistasis effect ρ detectable with a power 0.8 for each method for various cohort size. Model M_4 (alternative), and r_a = 1.0, r_b = 1.5 (main effect on SNP a only).

		ρ									
	f	0.15					0.30				
	method	IndOR	SHEsisEpi	dss	fastepi	gboost	IndOR	SHEsisEpi	dss	fastepi	gboost
r^2	$n = n_0 = n_1$										
0.0	500	>20	>20	>20	>20	>20	>20	>20	7.5	>20	>20
	1000	>20	>20	>20	>20	>20	>20	10.5	4.1	>20	>20
	2500	>20	>20	>20	>20	>20	5.2	4.3	>20	>20	>20
	5000	>20	>20	>20	>20	>20	3.2	2.8	>20	>20	>20
	10000	>20	5.2	>20	>20	>20	2.2	2.1	>20	>20	>20
0.2	500	>20	>20	>20	>20	>20	4.3	>20	6.6	>20	>20
	1000	>20	>20	14.1	>20	>20	2.5	12.9	3.8	>20	>20
	2500	2.9	>20	>20	>20	>20	1.4	5.2	>20	>20	15.2
	5000	1.6	15.2	>20	>20	8.7	1.1	3.1	>20	>20	5.8
	10000	1.1	5.8	>20	>20	4.6	1.1	2.2	>20	>20	4.0
0.5	500	>20	>20	>20	>20	>20	3.5	>20	10.5	>20	>20
	1000	>20	>20	>20	>20	>20	2.2	14.1	4.9	>20	15.2
	2500	1.9	>20	>20	>20	10.5	1.4	4.3	>20	>20	6.3
	5000	1.1	10.5	>20	>20	5.5	1.1	2.5	>20	>20	4.0
	10000	1.1	4.3	>20	>20	3.5	1.1	1.7	>20	6.6	2.9

model M_4 : alternative with main effect on both SNPs

Table 12: Smallest epistasis effect ρ detectable with a power 0.8 for each method for various cohort size. Model M_4 (alternative), and r_a = 1.5, r_b = 1.5 (main effect on both SNPs).

		ρ									
	f	0.15					0.30				
	method	IndOR	SHEsisEpi	dss	fastepi	gboost	IndOR	SHEsisEpi	dss	fastepi	gboost
r^2	$n = n_0 = n_1$										
0.0	500	>20	>20	>20	>20	>20	>20	>20	5.8	>20	>20
	1000	>20	>20	>20	>20	>20	>20	11.7	3.4	>20	>20
	2500	>20	>20	>20	>20	>20	6.3	4.3	>20	>20	>20
	5000	>20	>20	>20	>20	>20	3.5	2.8	>20	>20	>20
	10000	>20	5.8	>20	>20	>20	2.4	2.1	>20	>20	>20
0.2	500	>20	>20	>20	>20	>20	2.8	>20	5.8	>20	>20
	1000	>20	>20	12.9	>20	>20	1.4	12.3	3.4	>20	>20
	2500	1.1	>20	>20	>20	>20	1.1	4.6	>20	>20	>20
	5000	1.1	15.2	>20	>20	15.2	1.1	2.8	>20	>20	10.5
	10000	1.1	5.8	>20	>20	6.9	1.1	2.0	>20	>20	6.0
0.5	500	>20	>20	>20	>20	>20	2.2	>20	8.7	>20	>20
	1000	>20	>20	>20	>20	>20	1.1	15.2	4.6	>20	>20
	2500	1.1	>20	>20	>20	>20	1.1	4.0	>20	>20	9.9
	5000	1.1	10.5	>20	>20	9.3	1.1	2.4	>20	>20	6.3
	10000	1.1	4.0	>20	>20	5.5	1.1	1.7	>20	>20	4.7

3 Influence of MAF

3.1 model M_1: dominant-dominant

3.1.1 model M_1 : dominant-dominant with no main effect

Table 13: Smallest epistasis effect ρ detectable with a power 0.8 for each method depending on the MAF of causal SNPs ($f_a = f_b = f$). Model M_1 (dominant-dominant), $n_0 = n_1 = 1000$,and $r_a = 1.0$, $r_b = 1.0$ (no main effect).

r^2	method<br>f	ρ<br>IndOR	SHEsisEpi	dss	fastepi	gboost
0.0	0.0200	>20	>20	>20	>20	>20
	0.0500	>20	>20	>20	>20	19.7
	0.1000	>20	10.5	6.6	>20	5.5
	0.1500	>20	5.5	4.2	10.5	4.3
	0.1750	>20	4.6	3.7	10.5	3.7
	0.2000	>20	4.3	3.4	10.5	3.7
	0.2250	>20	4.3	3.2	15.2	3.7
	0.2500	>20	4.0	3.1	>20	4.0
	0.2625	10.5	4.6	2.9	>20	4.0
	0.2750	6.9	4.3	2.9	>20	4.3
	0.3000	4.6	4.6	2.8	>20	4.6
	0.3250	4.0	4.6	2.8	>20	5.2
	0.3500	4.0	5.2	2.8	>20	5.5
	0.3750	4.0	5.8	2.8	>20	6.9
	0.4000	5.2	8.1	2.8	>20	10.5
	0.4250	5.8	10.5	2.8	>20	>20
	0.4500	>20	>20	2.9	>20	>20
	0.5000	>20	>20	3.1	>20	>20
	0.5500	>20	>20	3.4	>20	>20
	0.6000	>20	>20	3.7	>20	>20
	0.7000	>20	>20	6.9	>20	>20
	0.7500	>20	>20	15.2	>20	>20
	0.8000	>20	>20	>20	>20	>20
	0.9000	>20	>20	>20	>20	>20
0.2	0.0200	>20	>20	>20	>20	>20
	0.0500	>20	18.8	11.7	>20	10.5
	0.1000	>20	8.1	5.5	>20	8.1
	0.2000	3.1	5.5	3.7	>20	18.8
	0.4000	2.2	>20	3.7	>20	>20
	0.5000	2.6	>20	4.2	>20	>20
0.5	0.0200	>20	>20	>20	>20	>20
	0.0500	>20	>20	>20	>20	>20
	0.1000	>20	18.8	9.3	>20	>20
	0.2000	2.4	15.2	5.8	12.9	>20
	0.4000	2.2	>20	5.8	10.5	8.7
	0.5000	2.5	>20	8.1	>20	6.9

3.1.2 model M_1 : dominant-dominant with main effect on SNP a only

Table 14: Smallest epistasis effect ρ detectable with a power 0.8 for each method depending on the MAF of causal SNPs ($f_a = f_b = f$). Model M_1 (dominant-dominant), $n_0 = n_1 = 1000$,and $r_a = 1.0$, $r_b = 1.5$ (main effect on SNP a only).

r^2	method<br>f	ρ<br>IndOR	SHEsisEpi	dss	fastepi	gboost
0.0	0.02	>20	>20	>20	>20	>20
	0.05	>20	>20	>20	>20	>20
	0.10	>20	12.9	9.3	>20	6.0
	0.20	>20	4.6	4.0	15.2	3.7
	0.40	6.9	>20	2.8	>20	11.7
	0.50	>20	>20	3.2	>20	>20
0.2	0.02	>20	>20	>20	>20	>20
	0.05	>20	>20	12.9	>20	10.5
	0.10	>20	8.1	5.8	>20	8.1
	0.20	2.0	5.8	3.7	>20	18.8
	0.40	1.8	>20	3.5	>20	>20
	0.50	2.5	>20	4.0	>20	>20
0.5	0.02	>20	>20	>20	>20	>20
	0.05	>20	>20	>20	>20	>20
	0.10	>20	>20	8.1	>20	>20
	0.20	1.5	17.6	5.8	6.9	>20
	0.40	1.6	>20	5.8	6.9	5.2
	0.50	1.8	>20	6.9	15.2	4.6

3.1.3 model M_1 : dominant-dominant with main effect on both SNPs

Table 15: Smallest epistasis effect ρ detectable with a power 0.8 for each method depending on the MAF of causal SNPs ($f_a = f_b = f$). Model M_1 (dominant-dominant), $n_0 = n_1 = 1000$,and $r_a = 1.5$, $r_b = 1.5$ (main effect on both SNPs).

r^2	method f	ρ IndOR	SHEsisEpi	dss	fastepi	gboost
0.0	0.02	>20	>20	>20	>20	>20
	0.05	>20	>20	>20	>20	>20
	0.10	>20	>20	6.3	>20	5.8
	0.20	>20	4.6	2.5	15.2	3.6
	0.40	>20	>20	1.8	>20	10.5
	0.50	>20	>20	2.0	>20	>20
0.2	0.02	>20	>20	>20	>20	>20
	0.05	>20	>20	9.3	>20	11.7
	0.10	>20	9.3	4.3	>20	8.1
	0.20	1.2	6.0	2.7	>20	>20
	0.40	1.4	>20	2.5	>20	>20
	0.50	2.3	>20	2.8	>20	>20
0.5	0.02	>20	>20	>20	>20	>20
	0.05	>20	>20	18.8	>20	>20
	0.10	>20	17.6	5.8	>20	>20
	0.20	1.1	18.8	4.0	4.6	>20
	0.40	1.1	>20	4.1	4.6	3.4
	0.50	1.4	>20	5.8	10.5	2.8

3.2 model M_2: recessive-recessive

3.2.1 model M_2 : recessive-recessive with no main effect

Table 16: Smallest epistasis effect ρ detectable with a power 0.8 for each method depending on the MAF of causal SNPs ($f_a = f_b = f$). Model M_2 (recessive-recessive), $n_0 = n_1 = 1000$,and $r_a = 1.0$, $r_b = 1.0$ (no main effect).

r^2	method f	ρ IndOR	SHEsisEpi	dss	fastepi	gboost
0.0	0.100	>20	>20	>20	>20	>20
	0.200	>20	>20	>20	>20	>20
	0.300	>20	>20	>20	>20	>20
	0.350	>20	>20	>20	>20	>20
	0.375	>20	>20	15.2	>20	>20
	0.400	>20	>20	9.9	>20	>20
	0.425	>20	11.1	7.5	15.2	12.9
	0.450	13.5	8.1	6.3	10.5	9.3
	0.475	11.7	6.9	5.2	9.3	8.1
	0.500	9.3	6.0	4.9	8.1	6.9
	0.525	8.1	5.5	4.6	6.6	5.9
	0.550	6.9	4.9	3.8	5.8	5.2
	0.575	7.2	4.7	3.7	5.5	4.9
	0.600	6.3	4.6	3.4	5.2	4.6
	0.625	6.0	4.6	3.2	5.2	4.6
	0.650	5.8	4.3	3.1	4.6	4.0
	0.700	5.8	4.6	2.9	4.6	4.0
	0.800	>20	8.1	2.8	8.1	5.8
	0.900	>20	>20	3.4	>20	>20
0.2	0.100	>20	>20	>20	>20	>20
	0.200	>20	>20	>20	>20	>20
	0.400	5.8	8.1	6.3	5.5	4.9
	0.500	3.8	5.2	4.4	4.0	3.5
0.5	0.100	>20	>20	>20	>20	>20
	0.200	10.5	>20	>20	18.8	10.5
	0.400	3.2	8.1	8.1	4.0	3.6
	0.500	2.6	6.3	6.3	3.4	3.1

3.2.2 model M_2 : recessive-recessive with main effect on SNP a only

Table 17: Smallest epistasis effect ρ detectable with a power 0.8 for each method depending on the MAF of causal SNPs ($f_a = f_b = f$). Model M_2 (recessive-recessive), $n_0 = n_1 = 1000$,and $r_a = 1.0$, $r_b = 1.5$ (main effect on SNP a only).

r^2	method f	ρ IndOR	SHEsisEpi	dss	fastepi	gboost
0.0	0.1	>20	>20	>20	>20	>20
	0.2	>20	>20	>20	>20	>20
	0.4	>20	>20	11.4	>20	>20
	0.5	10.5	5.8	4.9	7.5	6.9
0.2	0.1	>20	>20	>20	>20	>20
	0.2	>20	>20	>20	>20	>20
	0.4	4.0	5.8	6.3	7.1	6.9
	0.5	3.4	4.6	4.3	4.9	4.6
0.5	0.1	>20	>20	>20	>20	>20
	0.2	5.2	>20	>20	>20	>20
	0.4	2.5	5.2	9.3	6.0	5.5
	0.5	2.2	4.6	6.3	4.3	4.6

3.2.3 model M_2 : recessive-recessive with main effect on both SNPs

Table 18: Smallest epistasis effect ρ detectable with a power 0.8 for each method depending on the MAF of causal SNPs ($f_a = f_b = f$). Model M_2 (recessive-recessive), $n_0 = n_1 = 1000$,and $r_a = 1.5$, $r_b = 1.5$ (main effect on both SNPs).

r^2	method<br>f	ρ<br>IndOR	SHEsisEpi	dss	fastepi	gboost
0.0	0.1	>20	>20	>20	>20	>20
	0.2	>20	>20	>20	>20	>20
	0.4	>20	>20	10.5	>20	>20
	0.5	9.3	5.8	4.6	8.1	8.1
0.2	0.1	>20	>20	>20	>20	>20
	0.2	3.1	>20	>20	>20	>20
	0.4	2.9	5.2	6.3	9.3	8.7
	0.5	2.8	4.0	4.6	5.8	5.8
0.5	0.1	>20	>20	>20	>20	>20
	0.2	1.3	>20	>20	>20	>20
	0.4	1.6	4.0	8.1	8.1	7.5
	0.5	1.7	4.0	6.3	5.8	6.3

3.3 model M_3: multiplicative

3.3.1 model M_3 : multiplicative with no main effect

Table 19: Smallest epistasis effect ρ detectable with a power 0.8 for each method depending on the MAF of causal SNPs ($f_a = f_b = f$). Model M_3 (multiplicative), $n_0 = n_1 = 1000$,and $r_a = 1.0$, $r_b = 1.0$ (no main effect).

r^2	method<br>f	ρ<br>IndOR	SHEsisEpi	dss	fastepi	gboost
0.0	0.020	>20	>20	>20	>20	>20
	0.050	>20	>20	>20	>20	17.6
	0.075	>20	>20	10.5	>20	6.3
	0.100	>20	8.7	5.8	>20	4.3
	0.125	>20	5.5	4.3	8.1	3.4
	0.150	>20	4.3	3.4	5.2	3.1
	0.175	>20	3.7	3.1	4.6	2.6
	0.200	>20	3.4	2.7	4.0	2.3
	0.225	>20	3.1	2.4	3.4	2.2
	0.250	>20	2.9	2.2	3.4	2.1
	0.275	>20	2.8	2.1	2.8	2.0
	0.300	>20	2.8	2.0	>20	2.0
	0.325	>20	2.8	1.9	>20	1.9
	0.350	>20	2.9	1.9	>20	1.9
	0.375	>20	3.1	1.8	>20	1.8
	0.400	>20	3.4	1.7	>20	1.8
	0.425	>20	10.5	1.7	>20	1.8
	0.450	>20	12.9	1.7	>20	1.8
	0.500	>20	>20	1.7	>20	>20
	0.600	>20	>20	1.7	>20	>20
	0.700	>20	>20	1.7	>20	>20
	0.800	>20	>20	1.7	>20	>20
	0.900	>20	>20	1.8	>20	>20
0.2	0.020	>20	>20	>20	>20	>20
	0.050	>20	17.6	10.5	>20	9.3
	0.100	>20	6.3	4.4	>20	4.0
	0.200	>20	3.7	2.6	>20	2.3
	0.400	>20	>20	2.3	>20	>20
	0.500	>20	>20	2.3	>20	>20
0.5	0.020	>20	>20	>20	>20	>20
	0.050	>20	>20	>20	>20	17.6
	0.100	>20	17.6	7.5	>20	8.1
	0.200	>20	8.1	4.0	>20	2.3
	0.400	>20	>20	5.8	>20	>20
	0.500	>20	>20	3.4	>20	>20

3.3.2 model M_3 : multiplicative with main effect on SNP a only

Table 20: Smallest epistasis effect ρ detectable with a power 0.8 for each method depending on the MAF of causal SNPs ($f_a = f_b = f$). Model M_3 (multiplicative), $n_0 = n_1 = 1000$,and $r_a = 1.0$, $r_b = 1.5$ (main effect on SNP a only).

r^2	method<br>f	ρ<br>IndOR	SHEsisEpi	dss	fastepi	gboost
0.0	0.02	>20	>20	>20	>20	>20
	0.05	>20	>20	>20	>20	>20
	0.10	>20	10.5	8.1	>20	4.6
	0.20	>20	3.5	2.8	4.6	2.4
	0.40	>20	10.5	1.9	>20	1.9
	0.50	>20	>20	1.7	>20	>20
0.2	0.02	>20	>20	>20	>20	>20
	0.05	>20	>20	10.5	>20	8.1
	0.10	>20	6.9	4.6	>20	4.6
	0.20	>20	4.0	2.7	>20	2.8
	0.40	>20	>20	2.1	>20	>20
	0.50	>20	>20	2.1	>20	>20
0.5	0.02	>20	>20	>20	>20	>20
	0.05	>20	>20	>20	>20	17.6
	0.10	>20	17.6	8.1	>20	6.9
	0.20	>20	>20	3.7	>20	3.4
	0.40	>20	>20	5.8	>20	>20
	0.50	>20	>20	3.4	>20	>20

3.3.3 model M_3 : multiplicative with main effect on both SNPs

Table 21: Smallest epistasis effect ρ detectable with a power 0.8 for each method depending on the MAF of causal SNPs ($f_a = f_b = f$). Model M_3 (multiplicative), $n_0 = n_1 = 1000$,and $r_a = 1.5$, $r_b = 1.5$ (main effect on both SNPs).

r^2	method / f	ρ IndOR	SHEsisEpi	dss	fastepi	gboost
0.0	0.02	>20	>20	>20	>20	>20
	0.05	>20	>20	>20	>20	>20
	0.10	>20	>20	5.5	>20	4.6
	0.20	>20	3.7	2.0	5.8	2.4
	0.40	>20	10.5	1.5	>20	2.0
	0.50	>20	>20	1.4	>20	>20
0.2	0.02	>20	>20	>20	>20	>20
	0.05	>20	>20	9.3	>20	9.3
	0.10	>20	6.9	3.4	>20	4.6
	0.20	>20	4.3	2.0	>20	3.1
	0.40	>20	>20	1.6	>20	>20
	0.50	>20	>20	2.2	>20	>20
0.5	0.02	>20	>20	>20	>20	>20
	0.05	>20	>20	15.2	>20	18.8
	0.10	>20	16.4	5.8	>20	8.1
	0.20	>20	>20	2.8	>20	5.2
	0.40	>20	>20	4.6	>20	>20
	0.50	>20	>20	3.4	>20	>20

3.4 model M_4: alternative

3.4.1 model M_4 : alternative with no main effect

Table 22: Smallest epistasis effect ρ detectable with a power 0.8 for each method depending on the MAF of causal SNPs ($f_a = f_b = f$). Model M_4 (alternative), $n_0 = n_1 = 1000$,and $r_a = 1.0$, $r_b = 1.0$ (no main effect).

r^2	method<br>f	ρ<br>IndOR	SHEsisEpi	dss	fastepi	gboost
0.0	0.020	>20	>20	>20	>20	>20
	0.050	>20	>20	>20	>20	>20
	0.100	>20	>20	>20	>20	>20
	0.150	>20	>20	>20	>20	>20
	0.175	>20	>20	>20	>20	>20
	0.200	>20	>20	14.1	>20	>20
	0.225	>20	>20	9.3	>20	>20
	0.250	>20	>20	7.2	>20	>20
	0.275	>20	15.2	5.8	>20	>20
	0.325	15.2	9.3	4.6	>20	>20
	0.350	10.5	7.5	4.0	>20	>20
	0.400	8.1	5.8	3.4	>20	>20
	0.450	6.6	5.2	3.1	>20	>20
	0.500	5.8	4.6	2.8	14.1	19.7
	0.550	5.2	4.3	2.7	10.5	12.9
	0.600	5.5	4.0	2.6	8.7	8.7
	0.700	>20	4.6	2.9	6.9	5.8
	0.800	>20	6.9	4.6	15.2	5.8
	0.900	>20	>20	>20	>20	>20
0.2	0.020	>20	>20	>20	>20	>20
	0.050	>20	>20	>20	>20	>20
	0.100	>20	>20	>20	>20	>20
	0.200	9.3	>20	8.1	>20	>20
	0.400	3.2	9.3	3.5	>20	>20
	0.500	3.1	6.3	3.1	>20	>20
0.5	0.020	>20	>20	>20	>20	>20
	0.050	>20	>20	>20	>20	>20
	0.100	>20	>20	>20	>20	>20
	0.200	5.2	>20	12.9	>20	11.7
	0.400	2.6	10.5	4.6	>20	>20
	0.500	2.3	8.1	4.0	>20	>20

3.4.2 model M_4 : alternative with main effect on SNP a only

Table 23: Smallest epistasis effect ρ detectable with a power 0.8 for each method depending on the MAF of causal SNPs ($f_a = f_b = f$). Model M_4 (alternative), $n_0 = n_1 = 1000$,and $r_a = 1.0$, $r_b = 1.5$ (main effect on SNP a only).

r^2	method<br>f	ρ<br>IndOR	SHEsisEpi	dss	fastepi	gboost
0.0	0.02	>20	>20	>20	>20	>20
	0.05	>20	>20	>20	>20	>20
	0.10	>20	>20	>20	>20	>20
	0.20	>20	>20	10.5	>20	>20
	0.40	8.1	5.9	2.9	>20	>20
	0.50	5.8	4.9	2.6	14.1	>20
0.2	0.02	>20	>20	>20	>20	>20
	0.05	>20	>20	>20	>20	>20
	0.10	>20	>20	>20	>20	>20
	0.20	4.0	>20	6.9	>20	>20
	0.40	2.5	8.7	3.0	>20	>20
	0.50	2.6	5.8	2.8	>20	>20
0.5	0.02	>20	>20	>20	>20	>20
	0.05	>20	>20	>20	>20	>20
	0.10	>20	>20	>20	>20	>20
	0.20	3.1	>20	9.9	>20	>20
	0.40	2.0	8.7	4.0	>20	>20
	0.50	2.0	6.9	3.6	>20	>20

3.4.3 model M_4 : alternative with main effect on both SNPs

Table 24: Smallest epistasis effect ρ detectable with a power 0.8 for each method depending on the MAF of causal SNPs ($f_a = f_b = f$). Model M_4 (alternative), $n_0 = n_1 = 1000$,and $r_a = 1.5$, $r_b = 1.5$ (main effect on both SNPs).

r^2	method / f	ρ IndOR	SHEsisEpi	dss	fastepi	gboost
0.0	0.02	>20	>20	>20	>20	>20
	0.05	>20	>20	>20	>20	>20
	0.10	>20	>20	>20	>20	>20
	0.20	>20	>20	8.1	>20	>20
	0.40	9.3	5.8	2.6	>20	>20
	0.50	5.8	4.6	2.3	11.7	17.6
0.2	0.02	>20	>20	>20	>20	>20
	0.05	>20	>20	>20	>20	>20
	0.10	>20	>20	>20	>20	>20
	0.20	1.7	>20	6.0	>20	>20
	0.40	1.9	8.1	2.8	>20	>20
	0.50	2.3	5.2	2.5	17.6	18.8
0.5	0.02	>20	>20	>20	>20	>20
	0.05	>20	>20	>20	>20	>20
	0.10	>20	>20	>20	>20	>20
	0.20	1.1	>20	8.4	>20	>20
	0.40	1.4	8.1	3.4	>20	>20
	0.50	1.6	6.3	3.1	>20	>20

4 Epistasis networks detected in the WTCCC GWAS on T2D

We consider the gene epistasis network formed by the gene pairs detected in epistasis by at least one of the five methods gboost (gbst), IndOR (ndR), dss (dss), fastepi (fstp), and SHEsisEpi (SHssp). Figures 5 to 18 presents each of the 14 connected components of this network.

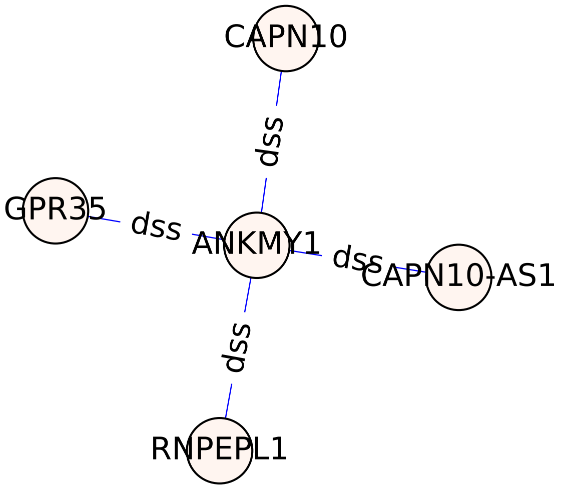

Figure 5: One connected component of the gene epistasis networks generated from all SNP pairs detected by one of the 5 methods. Node label indicates the genes detected in epistasis. A red node indicates that the gene was detected by univariate analysis. Edge label indicates the methods that detected a SNP pair in interaction between the connected genes: gboost (gbst), IndOR (ndR), dss (dss), fastepi (fstp), and SHEsisEpi (SHssp).

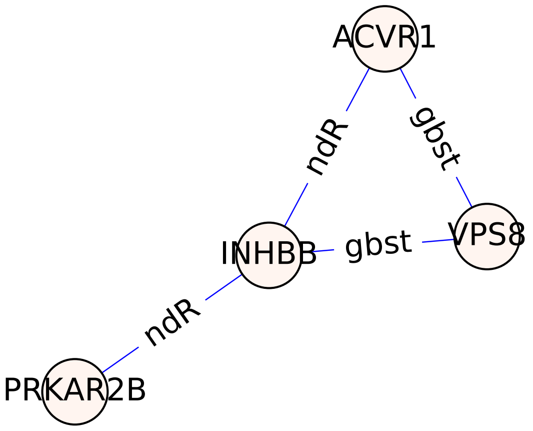

Figure 6: One connected component of the gene epistasis networks generated from all SNP pairs detected by one of the 5 methods. Node label indicates the genes detected in epistasis. A red node indicates that the gene was detected by univariate analysis. Edge label indicates the methods that detected a SNP pair in interaction between the connected genes: gboost (gbst), IndOR (ndR), dss (dss), fastepi (fstp), and SHEsisEpi (SHssp).

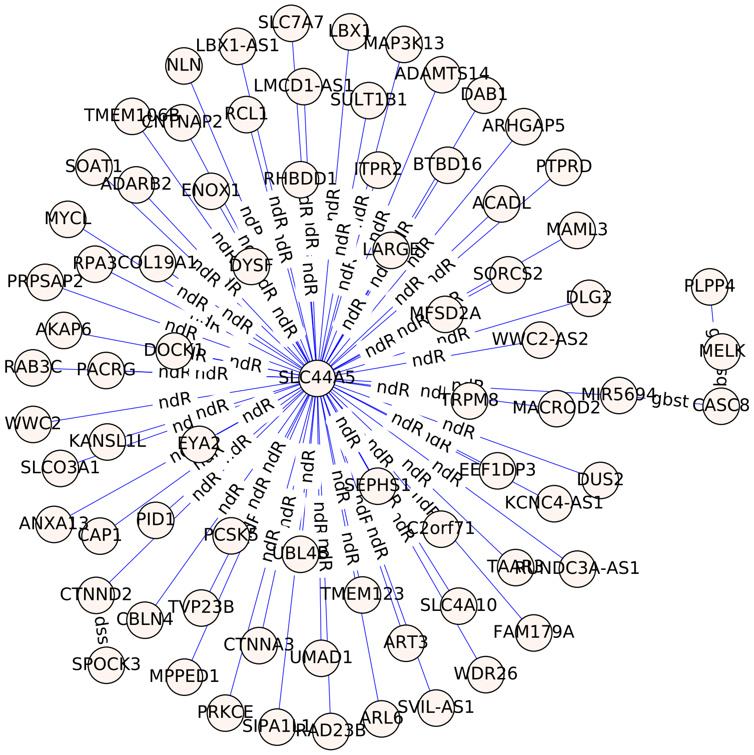

Figure 7: One connected component of the gene epistasis networks generated from all SNP pairs detected by one of the 5 methods. Node label indicates the genes detected in epistasis. A red node indicates that the gene was detected by univariate analysis. Edge label indicates the methods that detected a SNP pair in interaction between the connected genes: gboost (gbst), IndOR (ndR), dss (dss), fastepi (fstp), and SHEsisEpi (SHssp).

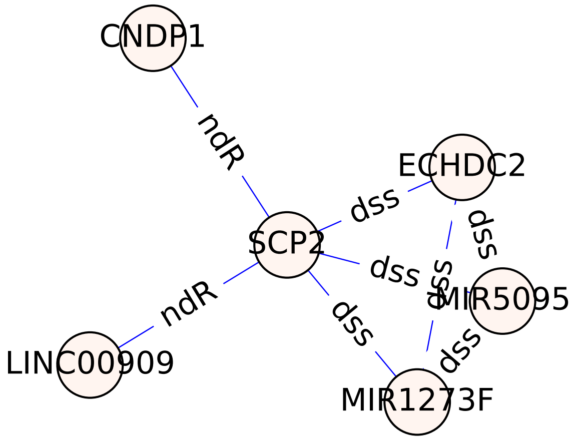

Figure 8: One connected component of the gene epistasis networks generated from all SNP pairs detected by one of the 5 methods. Node label indicates the genes detected in epistasis. A red node indicates that the gene was detected by univariate analysis. Edge label indicates the methods that detected a SNP pair in interaction between the connected genes: gboost (gbst), IndOR (ndR), dss (dss), fastepi (fstp), and SHEsisEpi (SHssp).

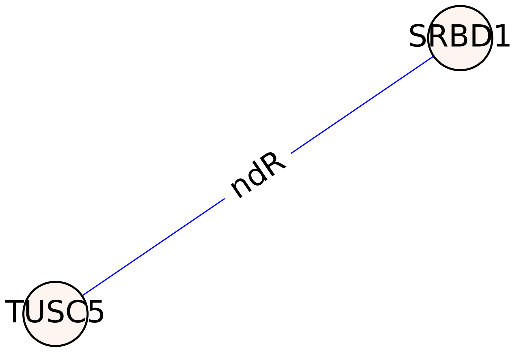

Figure 9: One connected component of the gene epistasis networks generated from all SNP pairs detected by one of the 5 methods. Node label indicates the genes detected in epistasis. A red node indicates that the gene was detected by univariate analysis. Edge label indicates the methods that detected a SNP pair in interaction between the connected genes: gboost (gbst), IndOR (ndR), dss (dss), fastepi (fstp), and SHEsisEpi (SHssp).

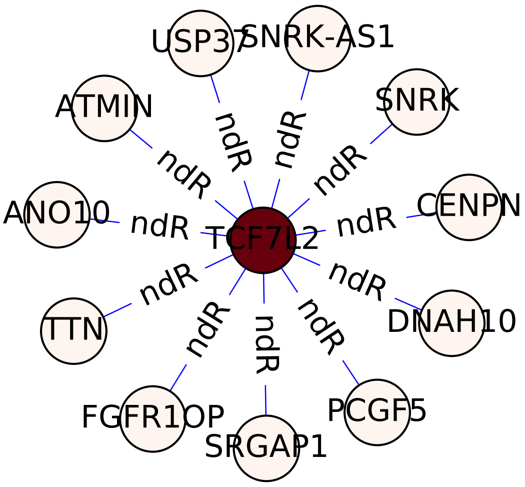

Figure 10: One connected component of the gene epistasis networks generated from all SNP pairs detected by one of the 5 methods. Node label indicates the genes detected in epistasis. A red node indicates that the gene was detected by univariate analysis. Edge label indicates the methods that detected a SNP pair in interaction between the connected genes: gboost (gbst), IndOR (ndR), dss (dss), fastepi (fstp), and SHEsisEpi (SHssp).

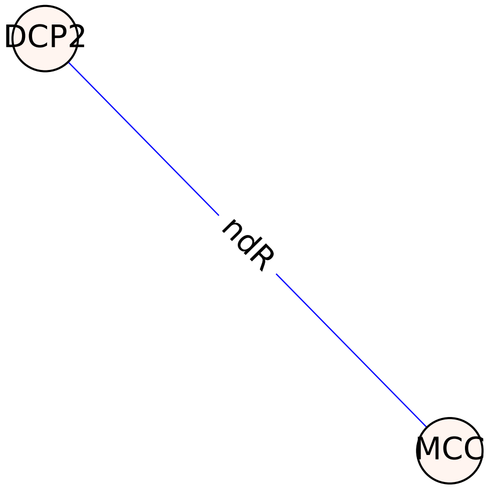

Figure 11: One connected component of the gene epistasis networks generated from all SNP pairs detected by one of the 5 methods. Node label indicates the genes detected in epistasis. A red node indicates that the gene was detected by univariate analysis. Edge label indicates the methods that detected a SNP pair in interaction between the connected genes: gboost (gbst), IndOR (ndR), dss (dss), fastepi (fstp), and SHEsisEpi (SHssp).

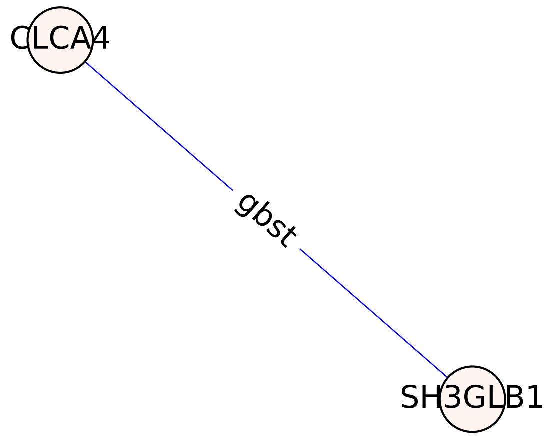

Figure 12: One connected component of the gene epistasis networks generated from all SNP pairs detected by one of the 5 methods. Node label indicates the genes detected in epistasis. A red node indicates that the gene was detected by univariate analysis. Edge label indicates the methods that detected a SNP pair in interaction between the connected genes: gboost (gbst), IndOR (ndR), dss (dss), fastepi (fstp), and SHEsisEpi (SHssp).

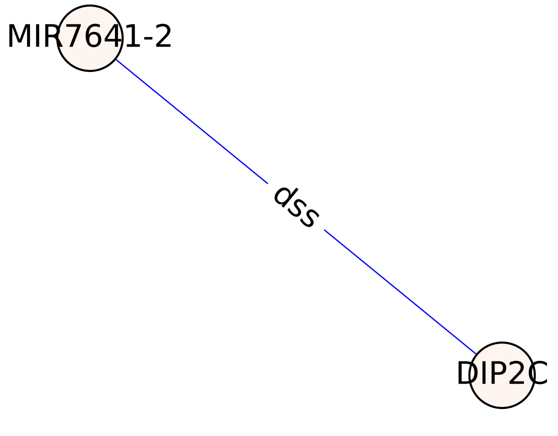

Figure 13: One connected component of the gene epistasis networks generated from all SNP pairs detected by one of the 5 methods. Node label indicates the genes detected in epistasis. A red node indicates that the gene was detected by univariate analysis. Edge label indicates the methods that detected a SNP pair in interaction between the connected genes: gboost (gbst), IndOR (ndR), dss (dss), fastepi (fstp), and SHEsisEpi (SHssp).

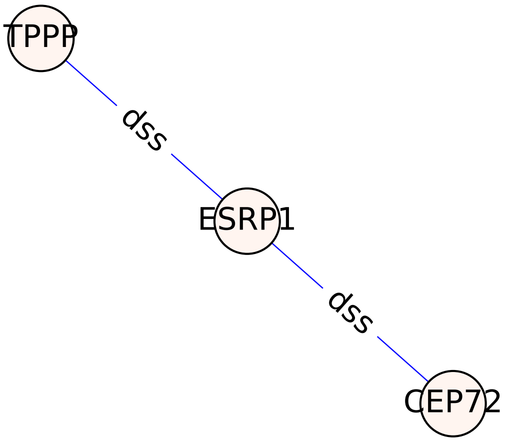

Figure 14: One connected component of the gene epistasis networks generated from all SNP pairs detected by one of the 5 methods. Node label indicates the genes detected in epistasis. A red node indicates that the gene was detected by univariate analysis. Edge label indicates the methods that detected a SNP pair in interaction between the connected genes: gboost (gbst), IndOR (ndR), dss (dss), fastepi (fstp), and SHEsisEpi (SHssp).

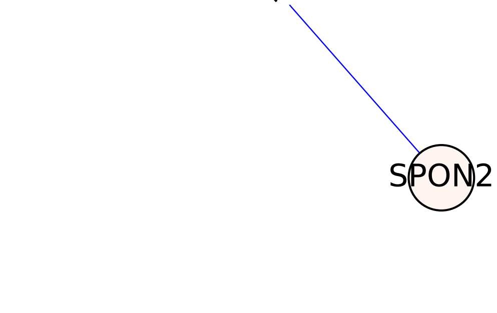

Figure 15: One connected component of the gene epistasis networks generated from all SNP pairs detected by one of the 5 methods. Node label indicates the genes detected in epistasis. A red node indicates that the gene was detected by univariate analysis. Edge label indicates the methods that detected a SNP pair in interaction between the connected genes: gboost (gbst), IndOR (ndR), dss (dss), fastepi (fstp), and SHEsisEpi (SHssp).

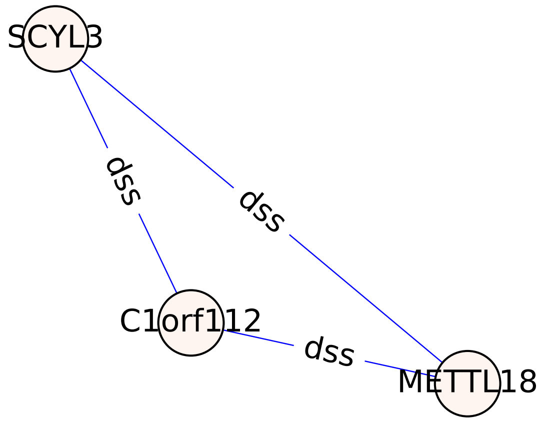

Figure 16: One connected component of the gene epistasis networks generated from all SNP pairs detected by one of the 5 methods. Node label indicates the genes detected in epistasis. A red node indicates that the gene was detected by univariate analysis. Edge label indicates the methods that detected a SNP pair in interaction between the connected genes: gboost (gbst), IndOR (ndR), dss (dss), fastepi (fstp), and SHEsisEpi (SHssp).

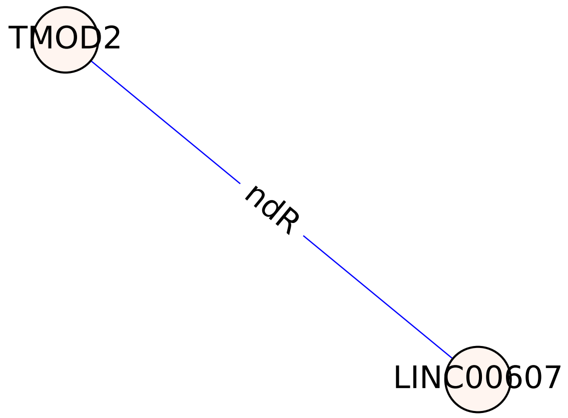

Figure 17: One connected component of the gene epistasis networks generated from all SNP pairs detected by one of the 5 methods. Node label indicates the genes detected in epistasis. A red node indicates that the gene was detected by univariate analysis. Edge label indicates the methods that detected a SNP pair in interaction between the connected genes: gboost (gbst), IndOR (ndR), dss (dss), fastepi (fstp), and SHEsisEpi (SHssp).

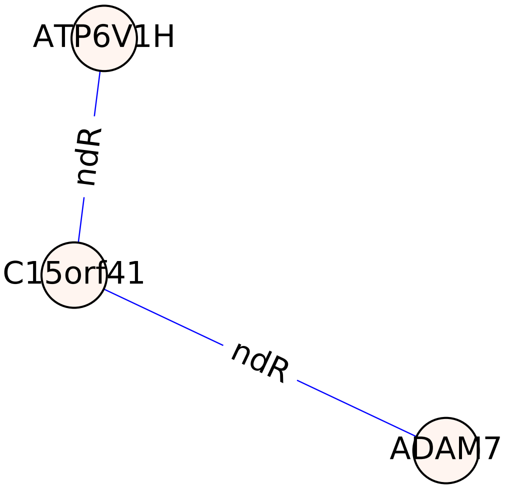

Figure 18: One connected component of the gene epistasis networks generated from all SNP pairs detected by one of the 5 methods. Node label indicates the genes detected in epistasis. A red node indicates that the gene was detected by univariate analysis. Edge label indicates the methods that detected a SNP pair in interaction between the connected genes: gboost (gbst), IndOR (ndR), dss (dss), fastepi (fstp), and SHEsisEpi (SHssp).
